# Polygenic risk scores and breast and epithelial ovarian cancer risks for carriers of *BRCA1* and *BRCA2* pathogenic variants

**DOI:** 10.1038/s41436-020-0862-x

**Published:** 2020-07-15

**Authors:** Daniel R. Barnes, Matti A. Rookus, Lesley McGuffog, Goska Leslie, Thea M. Mooij, Joe Dennis, Nasim Mavaddat, Julian Adlard, Munaza Ahmed, Kristiina Aittomäki, Nadine Andrieu, Irene L. Andrulis, Norbert Arnold, Banu K. Arun, Jacopo Azzollini, Judith Balmaña, Rosa B. Barkardottir, Daniel Barrowdale, Javier Benitez, Pascaline Berthet, Katarzyna Białkowska, Amie M. Blanco, Marinus J. Blok, Bernardo Bonanni, Susanne E. Boonen, Åke Borg, Aniko Bozsik, Angela R. Bradbury, Paul Brennan, Carole Brewer, Joan Brunet, Saundra S. Buys, Trinidad Caldés, Maria A. Caligo, Ian Campbell, Lise Lotte Christensen, Wendy K. Chung, Kathleen B. M. Claes, Chrystelle Colas, GEMO Study Collaborators, Pascaline Berthet, Chrystelle Colas, Marie-Agnès Collonge-Rame, Capucine Delnatte, Laurence Faivre, Sophie Giraud, Christine Lasset, Véronique Mari, Noura Mebirouk, Emmanuelle Mouret-Fourme, Hélène Schuster, Dominique Stoppa-Lyonnet, EMBRACE Collaborators, Julian Adlard, Munaza Ahmed, Antonis Antoniou, Daniel Barrowdale, Paul Brennan, Carole Brewer, Jackie Cook, Rosemarie Davidson, Douglas Easton, Ros Eeles, D. Gareth Evans, Debra Frost, Helen Hanson, Louise Izatt, Kai-ren Ong, Lucy Side, Aoife O’Shaughnessy-Kirwan, Marc Tischkowitz, Lisa Walker, Marie-Agnès Collonge-Rame, Jackie Cook, Mary B. Daly, Rosemarie Davidson, Miguel de la Hoya, Robin de Putter, Capucine Delnatte, Peter Devilee, Orland Diez, Yuan Chun Ding, Susan M. Domchek, Cecilia M. Dorfling, Martine Dumont, Ros Eeles, Bent Ejlertsen, Christoph Engel, D. Gareth Evans, Laurence Faivre, Lenka Foretova, Florentia Fostira, Michael Friedlander, Eitan Friedman, Debra Frost, Patricia A. Ganz, Judy Garber, Andrea Gehrig, Anne-Marie Gerdes, Paul Gesta, Sophie Giraud, Gord Glendon, Andrew K. Godwin, David E. Goldgar, Anna González-Neira, Mark H. Greene, Daphne Gschwantler-Kaulich, Eric Hahnen, Ute Hamann, Helen Hanson, Julia Hentschel, Frans B. L. Hogervorst, Maartje J. Hooning, Judit Horvath, Chunling Hu, Peter J. Hulick, Evgeny N. Imyanitov, kConFab Investigators, Georgia Chenevix-Trench, Kelly-Anne Phillips, Amanda Spurdle, HEBON Investigators, Marinus Blok, Peter Devilee, Frans Hogervorst, Maartje Hooning, Marco Koudijs, Arjen Mensenkamp, Hanne Meijers-Heijboer, Matti Rookus, Klaartje van Engelen, GENEPSO Investigators, Nadine Andrieu, Catherine Noguès, Claudine Isaacs, Louise Izatt, Angel Izquierdo, Anna Jakubowska, Paul A. James, Ramunas Janavicius, Esther M. John, Vijai Joseph, Beth Y. Karlan, Karin Kast, Marco Koudijs, Torben A. Kruse, Ava Kwong, Yael Laitman, Christine Lasset, Conxi Lazaro, Jenny Lester, Fabienne Lesueur, Annelie Liljegren, Jennifer T. Loud, Jan Lubiński, Phuong L. Mai, Siranoush Manoukian, Véronique Mari, Noura Mebirouk, Hanne E. J. Meijers-Heijboer, Alfons Meindl, Arjen R. Mensenkamp, Austin Miller, Marco Montagna, Emmanuelle Mouret-Fourme, Semanti Mukherjee, Anna Marie Mulligan, Katherine L. Nathanson, Susan L. Neuhausen, Heli Nevanlinna, Dieter Niederacher, Finn Cilius Nielsen, Liene Nikitina-Zake, Catherine Noguès, Edith Olah, Olufunmilayo I. Olopade, Kai-ren Ong, Aoife O’Shaughnessy-Kirwan, Ana Osorio, Claus-Eric Ott, Laura Papi, Sue K. Park, Michael T. Parsons, Inge Sokilde Pedersen, Bernard Peissel, Ana Peixoto, Paolo Peterlongo, Georg Pfeiler, Kelly-Anne Phillips, Karolina Prajzendanc, Miquel Angel Pujana, Paolo Radice, Juliane Ramser, Susan J. Ramus, Johanna Rantala, Gad Rennert, Harvey A. Risch, Mark Robson, Karina Rønlund, Ritu Salani, Hélène Schuster, Leigha Senter, Payal D. Shah, Priyanka Sharma, Lucy E. Side, Christian F. Singer, Thomas P. Slavin, Penny Soucy, Melissa C. Southey, Amanda B. Spurdle, Doris Steinemann, Zoe Steinsnyder, Dominique Stoppa-Lyonnet, Christian Sutter, Yen Yen Tan, Manuel R. Teixeira, Soo Hwang Teo, Darcy L. Thull, Marc Tischkowitz, Silvia Tognazzo, Amanda E. Toland, Alison H. Trainer, Nadine Tung, Klaartje van Engelen, Elizabeth J. van Rensburg, Ana Vega, Jeroen Vierstraete, Gabriel Wagner, Lisa Walker, Shan Wang-Gohrke, Barbara Wappenschmidt, Jeffrey N. Weitzel, Siddhartha Yadav, Xin Yang, Drakoulis Yannoukakos, Dario Zimbalatti, Kenneth Offit, Mads Thomassen, Fergus J. Couch, Rita K. Schmutzler, Jacques Simard, Douglas F. Easton, Georgia Chenevix-Trench, Antonis C. Antoniou, on behalf of the Consortium of Investigators of Modifiers of BRCA and BRCA2

**Affiliations:** 1 Centre for Cancer Genetic Epidemiology, Department of Public Health and Primary Care, University of Cambridge, Cambridge, UK; 2 The Netherlands Cancer Institute, Department of Epidemiology (PSOE), Amsterdam, The Netherlands; 3 Chapel Allerton Hospital, Yorkshire Regional Genetics Service, Leeds, UK; 4 Great Ormond Street Hospital for Children NHS Trust, North East Thames Regional Genetics Service, London, UK; 5 University of Helsinki, Department of Clinical Genetics, Helsinki University Hospital, Helsinki, Finland; 6 Inserm U900, Genetic Epidemiology of Cancer team, Paris, France; 7 Institut Curie, Paris, France; 8 Mines ParisTech, Fontainebleau, France; 9 Department of Life & Health Sciences, PSL University, Paris, France; 10 Lunenfeld-Tanenbaum Research Institute of Mount Sinai Hospital, Fred A. Litwin Center for Cancer Genetics, Toronto, ON Canada; 11 University of Toronto, Department of Molecular Genetics, Toronto, ON Canada; 12 University Hospital of Schleswig-Holstein, Campus Kiel, Christian-Albrechts University Kiel, Department of Gynaecology and Obstetrics, Kiel, Germany; 13 University Hospital of Schleswig-Holstein, Campus Kiel, Christian-Albrechts University Kiel, Institute of Clinical Molecular Biology, Kiel, Germany; 14 University of Texas MD Anderson Cancer Center, Department of Breast Medical Oncology, Houston, TX USA; 15 Fondazione IRCCS Istituto Nazionale dei Tumori di Milano, Unit of Medical Genetics, Department of Medical Oncology and Hematology, Milan, Italy; 16 Vall d’Hebron Institute of Oncology, High Risk and Cancer Prevention Group, Barcelona, Spain; 17 University Hospital of Vall d’Hebron, Department of Medical Oncology, Barcelona, Spain; 18 Landspitali University Hospital, Department of Pathology, Reykjavik, Iceland; 19 University of Iceland, BMC (Biomedical Centre), Faculty of Medicine, Reykjavik, Iceland; 20 Centro de Investigación en Red de Enfermedades Raras (CIBERER), Madrid, Spain; 21 Spanish National Cancer Research Centre (CNIO), Human Cancer Genetics Programme, Madrid, Spain; 22 Centre François Baclesse, Département de Biopathologie, Caen, France; 23 Pomeranian Medical University, Department of Genetics and Pathology, Szczecin, Poland; 24 University of California San Francisco, Cancer Genetics and Prevention Program, San Francisco, CA USA; 25 Maastricht University Medical Center, Department of Clinical Genetics, Maastricht, The Netherlands; 26 IEO, European Institute of Oncology IRCCS, Division of Cancer Prevention and Genetics, Milan, Italy; 27 Zealand University Hospital, Clinical Genetic Unit, Department of Paediatrics, Roskilde, Denmark; 28 Lund University, Division of Oncology and Pathology, Department of Clinical Sciences Lund, Lund, Sweden; 29 National Institute of Oncology, Department of Molecular Genetics, Budapest, Hungary; 30 Perelman School of Medicine at the University of Pennsylvania, Department of Medicine, Abramson Cancer Center, Philadelphia, PA USA; 31 Institute of Genetic Medicine, International Centre for Life, Northern Genetic Service, Newcastle upon Tyne, UK; 32 Royal Devon & Exeter Hospital, Department of Clinical Genetics, Exeter, UK; 33 ONCOBELL-IDIBELL-IDIBGI-IGTP, Catalan Institute of Oncology, CIBERONC, Hereditary Cancer Program, Barcelona, Spain; 34 Huntsman Cancer Institute, Department of Medicine, Salt Lake City, UT USA; 35 CIBERONC, Hospital Clinico San Carlos, IdISSC (Instituto de Investigación Sanitaria del Hospital Clínico San Carlos), Molecular Oncology Laboratory, Madrid, Spain; 36 University Hospital, SOD Genetica Molecolare, Pisa, Italy; 37 Peter MacCallum Cancer Center, Melbourne, VIC Australia; 38 The University of Melbourne, Sir Peter MacCallum Department of Oncology, Melbourne, VIC Australia; 39 Aarhus University Hospital, Department of Clinical Medicine,, Aarhus, Denmark; 40 Columbia University, Departments of Pediatrics and Medicine, New York, NY USA; 41 Ghent University, Centre for Medical Genetics, Ghent, Belgium; 42 Institut Curie, Service de Génétique, Paris, France; 43 CHU de Besançon, Service de Génétique, Besançon, France; 44 CHU Nantes, Laboratoire de genetique moleculaire, Nantes, France; 45 Centre Georges-François Leclerc, Unité d’oncogénétique, Centre de Lutte Contre le Cancer, Dijon, France; 46 Hospices Civils de Lyon, Department of Genetics, Bron, France; 47 Centre Léon Bérard, Unité de Prévention et d’Epidémiologie Génétique, Lyon, France; 48 Centre Antoine Lacassagne, Département d’Hématologie-Oncologie Médicale, Nice, France; 49 Unité d’Oncogénétique Centre de Lutte contre le Cancer Paul Strauss, Strasbourg, France; 50 Sheffield Children’s Hospital, Sheffield Clinical Genetics Service, Sheffield, UK; 51 Queen Elizabeth University Hospitals, Department of Clinical Genetics, Glasgow, UK; 52 The Institute of Cancer Research and Royal Marsden NHS Foundation Trust, Oncogenetics Team, London, UK; 53 The University of Manchester, Manchester Academic Health Science Centre, Manchester Universities Foundation Trust, St. Mary’s Hospital, Genomic Medicine, Division of Evolution and Genomic Sciences, Manchester, UK; 54 Manchester Academic Health Science Centre, Manchester Universities Foundation Trust, St. Mary’s Hospital, Genomic Medicine, North West Genomics hub, Manchester, UK; 55 Guy’s and St Thomas’ NHS Foundation Trust, Clinical Genetics, London, UK; 56 Birmingham Women’s Hospital Healthcare NHS Trust, West Midlands Regional Genetics Service, Birmingham, UK; 57 Princess Anne Hospital, Southampton, UK; 58 Cambridge University Hospitals NHS Foundation Trust, East Anglian Medical Genetics Service, Cambridge, UK; 59 McGill University, Program in Cancer Genetics, Departments of Human Genetics and Oncology, Montréal, QC Canada; 60 University of Cambridge, Department of Medical Genetics, National Institute for Health Research Cambridge Biomedical Research Centre, Cambridge, UK; 61 Oxford University Hospitals, Oxford Centre for Genomic Medicine, Oxford, UK; 62 Fox Chase Cancer Center, Department of Clinical Genetics, Philadelphia, PA USA; 63 Leiden University Medical Center, Department of Pathology, Leiden, The Netherlands; 64 Leiden University Medical Center, Department of Human Genetics, Leiden, The Netherlands; 65 Vall dHebron Institute of Oncology (VHIO), Oncogenetics Group, Barcelona, Spain; 66 University Hospital Vall dHebron, Clinical and Molecular Genetics Area, Barcelona, Spain; 67 Beckman Research Institute of City of Hope, Department of Population Sciences, Duarte, CA USA; 68 University of Pennsylvania, Basser Center for BRCA, Abramson Cancer Center, Philadelphia, PA USA; 69 University of Pretoria, Department of Genetics, Arcadia, South Africa; 70 Centre Hospitalier Universitaire de Québec – Université Laval Research Center, Genomics Center,, Québec City, QC Canada; 71 Rigshospitalet, Copenhagen University Hospital, Department of Oncology, Copenhagen, Denmark; 72 University of Leipzig, Institute for Medical Informatics, Statistics and Epidemiology, Leipzig, Germany; 73 DHU Dijon, Centre de Génétique, Dijon, France; 74 Masaryk Memorial Cancer Institute, Department of Cancer Epidemiology and Genetics, Brno, Czech Republic; 75 National Centre for Scientific Research ‘Demokritos’, Molecular Diagnostics Laboratory, INRASTES, Athens, Greece; 76 NHMRC Clinical Trials, ANZ GOTG Coordinating Centre, Camperdown, NSW Australia; 77 Chaim Sheba Medical Center, The Susanne Levy Gertner Oncogenetics Unit, Ramat Gan, Israel; 78 Tel Aviv University, Sackler Faculty of Medicine, Ramat Aviv, Israel; 79 Jonsson Comprehensive Cancer Centre, UCLA, Schools of Medicine and Public Health, Division of Cancer Prevention & Control Research, Los Angeles, CA USA; 80 Dana-Farber Cancer Institute, Cancer Risk and Prevention Clinic, Boston, MA USA; 81 University Würzburg, Department of Human Genetics, Würzburg, Germany; 82 Rigshospitalet, Copenhagen University Hospital, Department of Clinical Genetics, Copenhagen, Denmark; 83 CH Niort, Service Régional Oncogénétique Poitou-Charentes, Niort, France; 84 University of Kansas Medical Center, Department of Pathology and Laboratory Medicine, Kansas City, KS USA; 85 Huntsman Cancer Institute, University of Utah School of Medicine, Department of Dermatology, Salt Lake City, UT USA; 86 Clinical Genetics Branch, Division of Cancer Epidemiology and Genetics, National Cancer Institute, Bethesda, MD USA; 87 Medical University of Vienna, Dept of OB/GYN, Vienna, Austria; 88 Faculty of Medicine and University Hospital Cologne, University of Cologne, Center for Familial Breast and Ovarian Cancer, Cologne, Germany; 89 Faculty of Medicine and University Hospital Cologne, University of Cologne, Center for Integrated Oncology (CIO), Cologne, Germany; 90 German Cancer Research Center (DKFZ), Molecular Genetics of Breast Cancer, Heidelberg, Germany; 91 St George’s NHS Foundation Trust, Southwest Thames Regional Genetics Service, London, UK; 92 University Hospital Leipzig, Institute of Human Genetics, Leipzig, Germany; 93 The Netherlands Cancer Institute - Antoni van Leeuwenhoek hospital, Family Cancer Clinic, Amsterdam, The Netherlands; 94 Erasmus MC Cancer Institute, Department of Medical Oncology, Family Cancer Clinic, Rotterdam, The Netherlands; 95 University of Münster, Institute of Human Genetics, Münster, Germany; 96 Mayo Clinic, Department of Laboratory Medicine and Pathology, Rochester, MN USA; 97 NorthShore University HealthSystem, Center for Medical Genetics, Evanston, IL USA; 98 The University of Chicago Pritzker School of Medicine, Chicago, IL USA; 99 N.N. Petrov Institute of Oncology, St. Petersburg, Russia; 100 QIMR Berghofer Medical Research Institute, Department of Genetics and Computational Biology, Brisbane, QLD Australia; 101 The University of Melbourne, Department of Medicine, St Vincent’s Hospital, Fitzroy, VIC Australia; 102 The University of Melbourne, Centre for Epidemiology and Biostatistics, Melbourne School of Population and Global Health, Melbourne, VIC Australia; 103 University Medical Center Utrecht, Department of Medical Genetics, Utrecht, The Netherlands; 104 Radboud University Medical Center, Department of Human Genetics, Nijmegen, The Netherlands; 105 Amsterdam UMC, location AMC, Department of Clinical Genetics, Amsterdam, The Netherlands; 106 Amsterdam UMC, location VUmc, Department of Clinical Genetics, Amsterdam, The Netherlands; 107 Oncogénétique Clinique and Aix Marseille Univ, INSERM, IRD, SESSTIM, Institut Paoli-Calmettes, Département d’Anticipation et de Suivi des Cancers, Marseille, France; 108 Lombardi Comprehensive Cancer Center, Georgetown University, Washington, DC USA; 109 Pomeranian Medical University, Independent Laboratory of Molecular Biology and Genetic Diagnostics, Szczecin, Poland; 110 Peter MacCallum Cancer Center, Parkville Familial Cancer Centre, Melbourne, VIC Australia; 111 Vilnius University Hospital Santariskiu Clinics, Hematology, Oncology and Transfusion Medicine Center, Department of Molecular and Regenerative Medicine, Vilnius, Lithuania; 112 State Research Institute Centre for Innovative Medicine, Vilnius, Lithuania; 113 Stanford Cancer Institute, Stanford University School of Medicine, Department of Medicine, Division of Oncology, Stanford, CA USA; 114 Memorial Sloan-Kettering Cancer Center, Clinical Genetics Research Lab, Department of Cancer Biology and Genetics, New York, NY USA; 115 University of California at Los Angeles, David Geffen School of Medicine, Department of Obstetrics and Gynecology, Los Angeles, CA USA; 116 Cedars-Sinai Medical Center, Women’s Cancer Program at the Samuel Oschin Comprehensive Cancer Institute, Los Angeles, CA USA; 117 Department of Gynecology and Obstetrics, Medical Faculty and University Hospital Carl Gustav Carus, Technische Universität Dresden, Dresden, Germany; 118 Odense University Hospital, Department of Clinical Genetics, Odense, Denmark; 119 Cancer Genetics Centre, Hong Kong Hereditary Breast Cancer Family Registry, Happy Valley, Hong Kong; 120 The University of Hong Kong, Department of Surgery, Pok Fu Lam, Hong Kong; 121 Hong Kong Sanatorium and Hospital, Department of Surgery, Happy Valley, Hong Kong; 122 Lyon University, UMR CNRS 5558, Lyon, France; 123 Karolinska Institutet, Department of Oncology, Stockholm, Sweden; 124 Magee-Womens Hospital, University of Pittsburgh School of Medicine, Pittsburgh, PA USA; 125 University of Munich, Campus Großhadern, Department of Gynecology and Obstetrics, Munich, Germany; 126 Roswell Park Cancer Institute, NRG Oncology, Statistics and Data Management Center, Buffalo, NY USA; 127 Veneto Institute of Oncology IOV - IRCCS, Immunology and Molecular Oncology Unit, Padua, Italy; 128 Memorial Sloan-Kettering Cancer Center, Clinical Genetics Service, Department of Medicine, New York, NY USA; 129 University of Toronto, Department of Laboratory Medicine and Pathobiology, Toronto, ON Canada; 130 University Health Network, Laboratory Medicine Program, Toronto, ON Canada; 131 University of Helsinki, Department of Obstetrics and Gynecology, Helsinki University Hospital, Helsinki, Finland; 132 University Hospital Düsseldorf, Heinrich-Heine University Düsseldorf, Department of Gynecology and Obstetrics, Düsseldorf, Germany; 133 Rigshospitalet, Copenhagen University Hospital, Center for Genomic Medicine, Copenhagen, Denmark; 134 Latvian Biomedical Research and Study Centre, Riga, Latvia; 135 The University of Chicago, Center for Clinical Cancer Genetics, Chicago, IL USA; 136 Campus Virchov Klinikum, Charite, Institute of Human Genetics, Berlin, Germany; 137 University of Florence, Department of Experimental and Clinical Biomedical Sciences ‘Mario Serio’, Medical Genetics Unit, Florence, Italy; 138 Seoul National University College of Medicine, Department of Preventive Medicine, Seoul, Korea; 139 Seoul National University Graduate School, Department of Biomedical Sciences, Seoul, Korea; 140 Seoul National University, Cancer Research Institute, Seoul, Korea; 141 Aalborg University Hospital, Molecular Diagnostics, Aalborg, Denmark; 142 Aalborg University Hospital, Clinical Cancer Research Center, Aalborg, Denmark; 143 Aalborg University, Department of Clinical Medicine, Aalborg, Denmark; 144 Portuguese Oncology Institute, Department of Genetics, Porto, Portugal; 145 IFOM - the FIRC Institute of Molecular Oncology, Genome Diagnostics Program, Milan, Italy; 146 Medical University of Vienna, Dept of OB/GYN and Comprehensive Cancer Center, Vienna, Austria; 147 IDIBELL (Bellvitge Biomedical Research Institute), Catalan Institute of Oncology, ProCURE, Barcelona, Spain; 148 Fondazione IRCCS Istituto Nazionale dei Tumori (INT), Unit of Molecular Bases of Genetic Risk and Genetic Testing, Department of Research, Milan, Italy; 149 Klinikum rechts der Isar der Technischen Universität München, Department of Gynaecology and Obstetrics, Munich, Germany; 150 University of NSW Sydney, School of Women’s and Children’s Health, Faculty of Medicine, Sydney, NSW Australia; 151 The Kinghorn Cancer Centre, Garvan Institute of Medical Research, Sydney, NSW Australia; 152 University of NSW Sydney, Adult Cancer Program, Lowy Cancer Research Centre, Sydney, NSW Australia; 153 Karolinska Institutet, Clinical Genetics, Stockholm, Sweden; 154 Carmel Medical Center and Technion Faculty of Medicine, Clalit National Cancer Control Center, Haifa, Israel; 155 Yale School of Medicine, Chronic Disease Epidemiology, New Haven, CT USA; 156 Region of Southern Denmark, Vejle Hospital, Department of Clinical Genetics, Vejle, Denmark; 157 Wexner Medical Center, The Ohio State University, Department of Gynecology and Obstetrics, Columbus, OH USA; 158 Institut de Cancérologie Strasbourg Europe, ICANS, Strasbourg, France; 159 Université de Strasbourg, Laboratoire d’ImmunoRhumatologie Moléculaire, Plateforme GENOMAX, INSERM UMR_S 1109, LabEx TRANSPLANTEX, Fédération de Médecine Translationnelle de Strasbourg (FMTS), Faculté de Médecine, Strasbourg, France; 160 The Ohio State University, Clinical Cancer Genetics Program, Division of Human Genetics, Department of Internal Medicine, The Comprehensive Cancer Center, Columbus, OH USA; 161 University of Kansas Medical Center, Department of Internal Medicine, Division of Medical Oncology, Westwood, KS USA; 162 City of Hope, Clinical Cancer Genomics, Duarte, CA USA; 163 Monash University, Precision Medicine, School of Clinical Sciences at Monash Health, Clayton, VIC Australia; 164 The University of Melbourne, Department of Clinical Pathology, Melbourne, VIC Australia; 165 Cancer Council Victoria, Cancer Epidemiology Division, Melbourne, VIC Australia; 166 Hannover Medical School, Institute of Human Genetics, Hannover, Germany; 167 INSERM U830, Department of Tumour Biology, Paris, France; 168 Université Paris Descartes, Paris, France; 169 University Hospital Heidelberg, Institute of Human Genetics, Heidelberg, Germany; 170 University of Porto, Biomedical Sciences Institute (ICBAS), Porto, Portugal; 171 Cancer Research Malaysia, Breast Cancer Research Programme, Subang Jaya, Selangor Malaysia; 172 University of Malaya, Department of Surgery, Faculty of Medicine, Kuala Lumpur, Malaysia; 173 Magee-Womens Hospital, University of Pittsburgh School of Medicine, Department of Medicine, Pittsburgh, PA USA; 174 The Ohio State University, Department of Cancer Biology and Genetics, Columbus, OH USA; 175 University Of Melbourne, Department of Medicine, Melbourne, VIC Australia; 176 Beth Israel Deaconess Medical Center, Department of Medical Oncology, Boston, MA USA; 177 Centro de Investigación en Red de Enfermedades Raras (CIBERER), Madrid, Spain; 178 Fundación Pública Galega de Medicina Xenómica, Santiago de Compostela, Spain; 179 Instituto de Investigación Sanitaria de Santiago de Compostela (IDIS), Complejo Hospitalario Universitario de Santiago, SERGAS, Santiago de Compostela, Spain; 180 University Hospital Ulm, Department of Gynaecology and Obstetrics, Ulm, Germany; 181 Mayo Clinic, Department of Oncology, Rochester, MN USA; 182 Faculty of Medicine and University Hospital Cologne, University of Cologne, Center for Molecular Medicine Cologne (CMMC), Cologne, Germany; 183 Centre for Cancer Genetic Epidemiology, Department of Oncology, University of Cambridge, Cambridge, UK

**Keywords:** *BRCA1*/*2*, breast cancer, ovarian cancer, PRS, genetics

## Abstract

**Purpose:**

We assessed the associations between population-based polygenic risk scores (PRS) for breast (BC) or epithelial ovarian cancer (EOC) with cancer risks for *BRCA1* and *BRCA2* pathogenic variant carriers.

**Methods:**

Retrospective cohort data on 18,935 *BRCA1* and 12,339 *BRCA2* female pathogenic variant carriers of European ancestry were available. Three versions of a 313 single-nucleotide polymorphism (SNP) BC PRS were evaluated based on whether they predict overall, estrogen receptor (ER)–negative, or ER-positive BC, and two PRS for overall or high-grade serous EOC. Associations were validated in a prospective cohort.

**Results:**

The ER-negative PRS showed the strongest association with BC risk for *BRCA1* carriers (hazard ratio [HR] per standard deviation = 1.29 [95% CI 1.25–1.33], *P* = 3×10^−72^). For *BRCA2*, the strongest association was with overall BC PRS (HR = 1.31 [95% CI 1.27–1.36], *P* = 7×10^−50^). HR estimates decreased significantly with age and there was evidence for differences in associations by predicted variant effects on protein expression. The HR estimates were smaller than general population estimates. The high-grade serous PRS yielded the strongest associations with EOC risk for *BRCA1* (HR = 1.32 [95% CI 1.25–1.40], *P* = 3×10^−22^) and *BRCA2* (HR = 1.44 [95% CI 1.30–1.60], *P* = 4×10^−12^) carriers. The associations in the prospective cohort were similar.

**Conclusion:**

Population-based PRS are strongly associated with BC and EOC risks for *BRCA1*/*2* carriers and predict substantial absolute risk differences for women at PRS distribution extremes.

## INTRODUCTION

Pathogenic variants in *BRCA1* and *BRCA2* are associated with high risk of developing breast and ovarian cancers.^1,2^ A recent study of *BRCA1*/*2* carriers estimated the average risk of developing breast cancer by age 80 years to be 72% for *BRCA1* and 69% for *BRCA2* carriers.^2^ Corresponding ovarian cancer risks were 44% for *BRCA1* and 17% for *BRCA2* carriers. This and previous studies have demonstrated that cancer risks for *BRCA1*/*2* carriers increase with an increasing number of affected first- or second-degree relatives,^2^ suggesting genetic or other familial factors modify cancer risks for *BRCA1*/*2* carriers. Consistent with this observation, common breast and ovarian cancer susceptibility single-nucleotide polymorphisms (SNPs), identified through genome-wide association studies (GWAS) in the general population, have been shown to modify breast and ovarian cancer risks for *BRCA1*/*2* carriers.^3–7^

Polygenic risk scores (PRS) based on the combined effects of disease-associated SNPs, can lead to significant levels of breast and ovarian cancer risk stratification in the general population.^8,9^ It has also been demonstrated that PRS can result in large absolute risk differences of developing these cancers for *BRCA1*/*2* carriers.^10^ The largest study to date was a retrospective cohort study of 23,463 carriers using a PRS based on up to 88 breast cancer susceptibility SNPs and a PRS based on up to 17 ovarian cancer susceptibility SNPs.^10^

Recent population-based GWAS identified an additional 72 breast and 12 ovarian cancer susceptibility SNPs.^6,7,11^ Based on these data, PRS have been constructed that include SNPs associated at both genome-wide and sub-genome-wide significance levels. The best performing PRS for breast cancer includes 313 SNPs.^12^

It is therefore important to understand how the most recently developed breast and ovarian cancer PRS modify cancer risks for *BRCA1*/*2* carriers, as this information will be necessary for implementation studies to evaluate how their application influences cancer risk management for women with pathogenic variants in these genes. In this study, we used the largest sample of women with pathogenic *BRCA1*/*2* variants currently available to assess the associations between the most recently developed PRS with cancer risks for *BRCA1*/*2* carriers. We evaluated how these PRS associations vary with age, cancer family history, and *BRCA1*/*2* gene variant characteristics. We further validated the associations for the first time in a prospective cohort of carriers and investigated implications for cancer risk prediction.

## MATERIALS AND METHODS

### Retrospective cohort study participants

Study participants were enrolled through 63 studies from 29 countries contributing to the Consortium of Investigators of Modifiers of *BRCA1*/*2* (CIMBA).^13^ Eligibility was restricted to women who were ≥18 years old at recruitment and carried a pathogenic *BRCA1*/*2* variant. CIMBA collected information on year of birth, variant description, age at study recruitment and last follow-up, age at breast and ovarian cancer (including invasive ovarian, fallopian tube, or peritoneal) diagnosis, age/date at bilateral prophylactic mastectomy, and number of first- and second-degree relatives with breast or ovarian cancer. Related individuals were tracked through a unique family identifier. The majority of study participants were recruited through cancer genetics clinics and enrolled in regional or national research studies. Variants were categorized according to their predicted or known effect on cellular protein expression: class I included loss-of-function pathogenic variants expected to result in unstable or no protein; class II included variants likely to yield stable mutant proteins.^14^ Breast cancer pathology data were available from pathology reviews, tumor registry records, medical records or pathology records, and from tissue microarray immunohistochemical staining.^15^

The genotyping, quality control and imputation processes have been described in detail previously^6,7^ (brief description provided in supplement). The present study was restricted to carriers of *BRCA1*/*2* pathogenic variants of European ancestry, determined using genetic data and multidimensional scaling.^6,7^

### Breast cancer PRS

The methods for calculating the PRS are described in the Supplementary material. We evaluated three versions of the published breast cancer PRS based on the same 313 SNPs, with different weights optimized to predict the risk of overall (PRS_BC_), ER-negative (PRS_ER-_), or ER-positive (PRS_ER+_) breast cancer^12^ (Table S1).

The breast cancer PRS were standardized using the standard deviations (SDs) of the corresponding PRS in population-based controls. Therefore, the estimated hazard ratios (HRs) from this study are directly comparable with odds ratios (ORs) estimated from population-based data.^12^

### Epithelial ovarian cancer PRS

We constructed ovarian cancer PRS based on ovarian cancer susceptibility SNPs identified through GWAS.^7^ Two ovarian cancer PRS were constructed: one for all invasive epithelial ovarian cancer (EOC) using 30 SNPs (PRS_EOC_); and one for predicting high-grade serous (HGS) EOC using 22 SNPs (PRS_HGS_) (Supplementary material, Table S2). HGS is the predominant EOC histotype in *BRCA1*/*2* tumors.^16^

The PRS SDs in unaffected women in our sample were used to standardize PRS_EOC_ and PRS_HGS_.

### Associations between PRS and breast cancer risk

Associations between PRS and breast cancer risk for *BRCA1*/*2* carriers were assessed using the CIMBA retrospective cohort. Study participants were censored at the first of (1) breast cancer diagnosis, (2) ovarian cancer diagnosis, (3) risk-reducing bilateral mastectomy, (4) last follow-up, or (5) age 80 years. Participants with a first breast cancer diagnosis were considered affected. To account for nonrandom sampling with respect to disease status, associations were evaluated using weighted Cox regression.^17,18^ This involved assigning age- and disease-specific sampling weights, such that observed weighted age-specific incidences agreed with established incidences for *BRCA1*/*2* pathogenic variant carriers (Supplementary material).^19^

We assessed the associations between three breast cancer PRS with the risk of overall breast cancer, and separately with ER-positive or ER-negative breast cancer risk. Models were stratified by country and Ashkenazi Jewish ancestry and were adjusted for birth cohort and the first four ancestry informative principal components calculated separately by genotyping array (Supplementary material). We fitted models adjusting for family history of breast cancer in first- and second-degree relatives to determine whether cancer family history was a confounder of PRS associations. Family history was coded as no family history, or one relative, or two or more relatives diagnosed with breast cancer. Robust variances were calculated to account for the inclusion of related individuals by clustering on family membership. All models were fitted separately in *BRCA1* and *BRCA2* carriers.

We fitted separate models in which the PRS was assumed to be (1) continuous and (2) categorical based on PRS percentiles determined by the PRS distribution in unaffected carriers. We tested for variation in the association of the PRS by age by fitting Cox regression models in which the PRS was a time-varying covariate, with age as the time scale, that included a PRS main effect and a PRS-by-age interaction term. Heterogeneity in the associations across countries was assessed by fitting models with a PRS–country interaction term. A likelihood ratio test (LRT) was used to assess statistical significance of interaction terms by comparing the models with the interaction against a model without the interaction term (Supplementary material). Similarly, LRTs were used to compare the fit of nested models.

Previous studies have demonstrated that cancer risks for *BRCA1*/*2* carriers vary by pathogenic variant location or functional effect.^2,20^ To investigate whether the PRS associations varied by *BRCA1/2-*variant location, we fitted models that included a PRS by location interaction. Variants were grouped into regions by nucleotide position on the basis of previously reported differences in breast or ovarian cancer risks. *BRCA1* variants were grouped in three regions (5’ to c.2281, c.2282 to c.4071, and c.4072 to 3’).^20,21^ The *BRCA2* ovarian cancer cluster region (OCCR) was used to define the variant location groups.^20,22^ Two *BRCA2* OCCR definitions were used: “narrow” (5’ to c.3846, c.3847 to c.6275, c.6276 to 3’) and “wide” (5’ to c.2831, c.2832 to c.6401, c.6402 to 3’). We also investigated variation in PRS associations by the predicted variant effect on protein stability/expression (class I versus class II, defined above).

To assess the associations with ER-specific breast cancer risk, a similar censoring process was used except the event of interest was diagnosis of either ER-positive or ER-negative breast cancer. Affected carriers with the alternative ER status to the outcome of interest were censored at that diagnosis. Carriers with missing ER status were excluded from the analysis.

### Associations with epithelial ovarian cancer risk

The associations with EOC risk were evaluated following a similar process. However, women were censored at bilateral risk-reducing salpingo-oophorectomy (RRSO) rather than bilateral mastectomy. Carriers with a first ovarian cancer diagnosis were assumed to be affected in this analysis. We also fitted models that adjusted for family history of ovarian cancer in first- and second-degree relatives, coded as no family history, or one relative, or two or more relatives diagnosed with the disease.

The discriminatory ability of each PRS was assessed by Harrell’s C-statistic^23^ stratified by country and Ashkenazi Jewish ancestry and adjusted for birth cohort and principal components.^24^ Standard errors were estimated using 1000 bootstrap replications.

### Validation in prospective cohorts

The PRS associations were further evaluated using prospective cohort data. The prospective cohort included pathogenic variant carriers from the *BRCA1* and *BRCA2* Cohort Consortium (BBCC)^2^ and CIMBA^13^ who were unaffected at recruitment (informed consent and baseline questionnaire). The BBCC included data from the International *BRCA1*/*2* Carrier Cohort Study (IBCCS), Breast Cancer Family Registry (BCFR), and the Kathleen Cunningham Foundation Consortium for Research into Familial Breast Cancer (kConFab) (details in Supplementary material).^2^ Only women of European ancestry were included in the analysis. All prospective cohort participants were genotyped as part of the CIMBA effort described above. However, prospective analyses considered only the prospective follow-up period from the time at recruitment of each participant into the study. Thus, the analysis time considered in the prospective and retrospective analyses were completely distinct. Associations were evaluated using Cox regression, separately for *BRCA1* and *BRCA2* carriers. The censoring process and analysis are described in detail in the Supplementary material.

### Predicted age-specific cancer risks by PRS

Retrospective analysis HR estimates were used to predict age-specific absolute risks of developing breast and ovarian cancer by PRS percentiles following a previously published method.^25^ To ensure consistency with known cancer risks for *BRCA1*/*2* carriers, average age-specific cancer incidences were constrained over PRS percentile categories to agree with external estimates of cancer incidences for carriers^2^ (Supplementary material). We also calculated absolute breast cancer risks for carriers in the absence or presence of cancer family history and by *BRCA2* variant location, assuming external average cancer incidences by family history and variant location.^2^ The absolute risks were used to calculate 10-year cancer risks at each age by different PRS percentiles (Supplementary material).

### Ethics statement

All study participants provided written informed consent and participated in research or clinical studies at the host institute under ethically approved protocols. The studies and their approving institutes are listed as a separate online Supplement (Ethics Statement).

All statistical tests were two-sided. Retrospective and prospective cohort analyses were performed using R 3.5.1. Age-varying PRS and discrimination analyses were conducted using Stata 13.1 (Supplementary material).

## RESULTS

The CIMBA retrospective cohort consisted of 18,935 *BRCA1* carriers (9473 diagnosed with breast and 2068 with ovarian cancer) and 12,339 *BRCA2* carriers (6332 with breast and 718 with ovarian cancer, Table S3).

The SNPs included in the PRS were well imputed on both genotyping platforms (Supplementary material, Figs. S1, S2, Tables S1, S2). The average PRS were larger for women diagnosed with cancer, compared with unaffected carriers (Table S3), but the PRS SDs were similar in unaffected and affected carriers (Table S3).

### Associations with breast cancer risk

Table 1 shows the associations between PRS_BC_, PRS_ER-_, and PRS_ER+_ and overall breast cancer risk for carriers using the CIMBA retrospective cohort data. PRS_ER-_ yielded the strongest association for *BRCA1* carriers (per SD HR = 1.29, 95% CI = 1.25–1.33, *P* = 3×10^−72^). For *BRCA2* carriers, the strongest associations were found for PRS_BC_ (per SD HR = 1.31, 95% CI = 1.27–1.36, *P* = 7×10^−50^) and PRS_ER+_ (per SD HR = 1.31, 95% CI = 1.26–1.36, *P* = 6×10^−49^). Adjusting for breast cancer family history yielded similar associations between the PRS and breast cancer risk to those observed in the unadjusted models (Table 1). Family history was significantly associated with risk in all models.

**Table 1:** PRS associations with breast and ovarian cancer risks for *BRCA1* and *BRCA2* pathogenic variant carriers using the CIMBA retrospective cohort data.

		*BRCA1* carriers					*BRCA2* carriers				
			No FH^a^ adjustment		FH adjusted			No FH adjustment		FH adjusted	
Outcome	PRS	Unaffected/ affected	HR (95% CI)	*P*	HR (95% CI)	*P*	Unaffected/ affected	HR (95% CI)	*P*	HR (95% CI)	*P*
Breast cancer	BC	9462/ 9473	1.20 (1.17–1.23)	1.15×10^−39^	1.20 (1.17–1.23)	9.54×10^−40^	6007/ 6332	**1.31 (1.27–1.36)**	**7.11×10**^**−50**^	**1.31 (1.26–1.36)**	**6.54×10**^**−48**^
	ER-		**1.29 (1.25–1.33)**	**3.03×10**^**−72**^	**1.29 (1.25–1.33)**	**1.02×10**^**−71**^		1.23 (1.19–1.28)	4.06×10^−29^	1.23 (1.18–1.27)	6.72×10^−28^
	ER+		1.17 (1.14–1.20)	6.93×10^−29^	1.17 (1.14–1.20)	5.50×10^−29^		1.31 (1.26–1.36)	6.12×10^−49^	1.30 (1.26–1.35)	5.10×10^−47^
ER-negative breast cancer	BC	10,138/ 3263	1.09 (1.05–1.13)	3.69×10^−6^	1.09 (1.05–1.13)	4.44×10^−6^	8049/ 703	1.20 (1.11–1.30)	4.90×10^−6^	1.19 (1.10–1.29)	1.91×10^−5^
	ER-		**1.23 (1.18–1.28)**	**2.39×10**^**−27**^	**1.23 (1.18–1.27)**	**1.08×10**^**−26**^		**1.31 (1.21–1.43)**	**1.15×10**^**−10**^	**1.29 (1.19–1.41)**	**9.98×10**^**−10**^
	ER+		1.06 (1.02–1.10)	4.58×10^−3^	1.06 (1.02–1.10)	4.93×10^−3^		1.17 (1.08–1.26)	1.36×10^−4^	1.15 (1.07–1.25)	3.91×10^−4^
ER-positive breast cancer	BC	12,376/ 1025	**1.44 (1.35–1.53)**	**3.88×10**^**−28**^	**1.44 (1.35–1.54)**	**1.25×10**^**−27**^	6440/ 2312	1.37 (1.31–1.44)	2.95×10^−40^	1.36 (1.30–1.43)	6.28×10^−38^
	ER-		1.29 (1.21–1.38)	2.94×10^−15^	1.29 (1.21–1.37)	9.25×10^−15^		1.22 (1.16–1.28)	1.93×10^−15^	1.21 (1.15–1.27)	1.54×10^−14^
	ER+		1.44 (1.35–1.54)	3.94×10^−28^	1.45 (1.35–1.54)	1.12×10^−27^		**1.38 (1.32–1.45)**	**1.88×10**^**−42**^	**1.37 (1.31–1.44)**	**5.99×10**^**−40**^
Ovarian cancer	EOC	16,867/ 2068	1.31 (1.24–1.39)	1.49×10^−21^	1.31 (1.24–1.39)	2.36×10^−21^	11,621/ 718	1.43 (1.29–1.59)	1.81×10^−11^	1.42 (1.28–1.58)	3.40×10^−11^
	HGS		**1.32 (1.25–1.40)**	**3.01×10**^**−22**^	**1.32 (1.25–1.40)**	**5.18×10**^**−22**^		**1.44 (1.30–1.60)**	**4.34×10**^**−12**^	**1.43 (1.29–1.59)**	**7.54×10**^**−12**^

*BC* breast cancer, *CI* confidence interval, *CIMBA* Consortium of Investigators of Modifiers of *BRCA1/2*, *ER-* estrogen receptor negative, *ER*+ estrogen receptor positive, *EOC* epithelial ovarian cancer, *FH* family history, *HGS* high-grade serous, *HR* hazard ratio, *PRS*polygenic risk score.

Rows in bold represent the best performing PRS for each particular outcome.

^a^Family history in first- and second-degree relatives: coded as no family history, or one relative, or two or more relatives diagnosed with the disease.

**Fig. 1:**
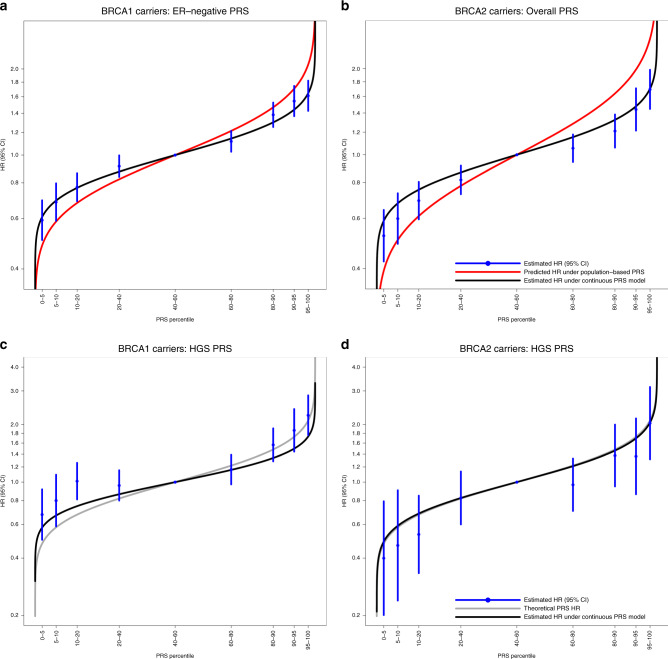
Associations with specific polygenic risk score (PRS) percentiles. The PRS percentile thresholds were determined in the sets of unaffected carriers for the disease under assessment. Table 2 shows the estimated hazard ratios (HRs). The black curve represents the expected HRs assuming the per standard deviation HR estimates in *BRCA1* and *BRCA2* carriers based on the continuous PRS models (Table 1). (**a**) PRS_ER-_ percentile-specific associations with breast cancer risk for *BRCA1* carriers. The red curve represents the expected HRs over the PRS percentile distribution, assuming the per SD odds ratio (OR) estimate from the population-based validation studies from Mavaddat et al.^12^ (OR = 1.45 per PRS_ER-_ standard deviation). (**b**) PRS_BC_ percentile-specific associations with breast cancer risk for *BRCA2* carriers. The red curve represents the expected HRs over the PRS percentile distribution, assuming the per SD OR estimate from the population-based validation studies from Mavaddat et al.^12^ (OR = 1.61 per PRS_BC_ standard deviation). (**c**) PRS_HGS_ percentile-specific associations with ovarian cancer risk for *BRCA1* carriers. (**d**) PRS_HGS_ percentile-specific associations with ovarian cancer risk for *BRCA2* carriers. The gray curve (**c** and **d** only) represents the theoretical HRs across the PRS distribution, calculated by assuming external single-nucleotide polymorphism (SNP) effect sizes and allele frequencies for SNPs contributing to the PRS. *CI* confidence interval, *ER* estrogen receptor, *HGS* high-grade serous.

The PRS_ER-_ and PRS_BC_ were used for subsequent *BRCA1* and *BRCA2* carrier analyses, respectively. There was no statistically significant evidence of heterogeneity in the country-specific HR estimates (*BRCA1* P_LRT_ = 0.26, *BRCA2* P_LRT_ = 0.64; Fig. S3). The estimated HRs for each PRS percentile category (Table 2) were consistent with the HRs predicted under models with the continuous PRS (estimated above), but were attenuated compared to the HRs expected under the population-based PRS distributions (Fig. 1a, b). Models estimating PRS percentile-specific associations did not fit significantly better than models in which PRS were continuous (*BRCA1* carriers P_LRT_ = 0.18; *BRCA2* carriers P_LRT_ = 0.99). The HRs for the breast cancer association decreased with age (Table 2; PRS-by-age interaction HRs: *BRCA1* HR = 0.996, *P* = 0.003; *BRCA2* HR = 0.994, *P* = 9.40×10^−5^). The HRs for the PRS associations with breast cancer risk did not differ by variant location (Table 2: *BRCA1* P_LRT_ = 0.17; *BRCA2* P_LRT_ ≥ 0.27). However, the associations differed by the predicted effect of the gene variant on protein stability/expression: the HRs for the PRS associations with breast cancer risk were larger for carriers with class II (stable mutant proteins) versus class I (unstable/no protein) variants (Table 2, *BRCA1*: class I HR = 1.26 [95% CI = 1.22–1.30], class II HR = 1.38 [1.30–1.46], P_difference_ = 0.011; *BRCA2*: class I HR = 1.30 [95% CI = 1.25–1.35], class II HR = 1.72 [95% CI = 1.44–2.06], P_difference_ = 0.003).

**Table 2:** Categorical PRS, age-varying and pathogenic variant characteristic specific PRS associations with cancer risks for *BRCA1* and *BRCA2* carriers, using data from the CIMBA retrospective cohort.

		Breast cancer						Ovarian cancer					
		*BRCA1* carriers: PRS_ER-_			*BRCA2* carriers: PRS_BC_			*BRCA1* carriers: PRS_HGS_			*BRCA2* carriers: PRS_HGS_		
Model	Category	HR (95% CI)	*P*	P_LRT_	HR (95% CI)	*P*	P_LRT_	HR (95% CI)	*P*	P_LRT_	HR (95% CI)	*P*	P_LRT_
Categorical PRS percentiles (%)	0–5	0.59 (0.50–0.70)			0.52 (0.42–0.64)			0.68 (0.50–0.92)			0.40 (0.20–0.79)		
	5–10	0.69 (0.59–0.80)			0.60 (0.49–0.73)			0.80 (0.59–1.09)			0.47 (0.24–0.91)		
	10–20	0.77 (0.69–0.86)			0.69 (0.59–0.80)			1.01 (0.81–1.26)			0.53 (0.33–0.85)		
	20–40	0.91 (0.84–1.00)			0.82 (0.73–0.92)			0.96 (0.80–1.15)			0.83 (0.60–1.14)		
	40–60	1.00 [reference]			1.00 [reference]			1.00 [reference]			1.00 [reference]		
	60–80	1.12 (1.03–1.21)			1.05 (0.94–1.18)			1.16 (0.97–1.39)			0.97 (0.71–1.33)		
	80–90	1.38 (1.25–1.53)			1.21 (1.06–1.38)			1.57 (1.28–1.91)			1.38 (0.95–2.00)		
	90–95	1.55 (1.37–1.75)			1.44 (1.21–1.71)			1.86 (1.44–2.41)			1.36 (0.86–2.15)		
	95–100	1.61 (1.43–1.82)			1.69 (1.45–1.98)			2.24 (1.76–2.84)			2.03 (1.31–3.15)		
Age-varying PRS^a^: model including a main PRS effect and a PRS × age interaction term	PRS	1.517 (1.359–1.694)	1.04×10^−13^	0.017	1.721 (1.498–1.977)	1.75×10^−14^	2.27×10^−3^	1.507 (1.125–2.020)	6.02×10^−3^	0.41	2.183 (1.263–3.774)	5.17×10^−3^	0.44
	PRS × age	0.996 (0.993–0.999)	3.27×10^−3^		0.994 (0.991–0.997)	9.40×10^−5^		0.997 (0.991–1.003)	0.35		0.992 (0.982–1.003)	0.14	
Gene pathogenic variant class	Class I	1.26 (1.22–1.30)	0.011^b^	5.29×10^−3^	1.30 (1.25–1.35)	3.20×10^−3 b^	0.046	1.33 (1.24–1.43)	0.85^b^	0.85	N/A^c^		
	Class II	1.38 (1.30–1.46)			1.72 (1.44–2.06)			1.32 (1.18–1.47)					
*BRCA1* pathogenic variant location	c.2282-c.4071	1.25 (1.19–1.31)		0.17	N/A			1.50 (1.35–1.66)		8.73×10^−3^	N/A		
	5’ to c.2281	1.28 (1.22–1.34)						1.30 (1.18–1.42)					
	c.4072 to 3′	1.34 (1.28–1.41)						1.21 (1.10–1.33)					
*BRCA2* pathogenic variant location (narrow)	c.3847-c.6275	N/A			1.30 (1.23–1.38)		0.27	N/A			1.48 (1.24–1.76)		0.96
	5’ to c.3846				1.26 (1.17–1.34)						1.41 (1.17–1.69)		
	c.6276 to 3′				1.37 (1.29–1.46)						1.43 (1.20–1.70)		
*BRCA2* pathogenic variant location (wide)	c.2831-c.6401	N/A			1.29 (1.23–1.36)		0.33	N/A			1.48 (1.26–1.75)		0.90
	5’ to c.2830				1.26 (1.17–1.37)						1.37 (1.13–1.68)		
	c.6402 to 3′				1.37 (1.29–1.46)						1.43 (1.20–1.71)		

Class I pathogenic variant refers to loss-of-function pathogenic variants expected to result in unstable or no protein; class II pathogenic variant refers to pathogenic variants likely to yield stable mutant proteins. *P* value for the Wald test statistic unless otherwise stated.LRT compares the models with an interaction term against the model without the interaction term.

*BC* breast cancer, *CI* confidence interval, *CIMBA* Consortium of Investigators of Modifiers of *BRCA1/2*, *ER-* estrogen receptor negative, *HGS* high-grade serous, *HR* hazard ratio, *LRT* likelihood ratio test, *N/A* not applicable.

^a^Age in years.

^b^*P* value for the difference in HR for class I carriers vs. the HR for class II carriers.

^c^Number of affected class II carriers was too small to make meaningful inference.

Under the age-varying PRS models, the C-statistic for PRS_ER-_ was 0.60 (95% CI = 0.59–0.61) for *BRCA1* carriers, and for the PRS_BC_ for *BRCA2* carriers 0.65 (95% CI = 0.63–0.67). Under models that did not include the age-varying PRS, the estimated C-statistics were 0.58 (95% CI = 0.57–0.59) and 0.60 (95% CI = 0.59–0.62) for *BRCA1* and *BRCA2* carriers, respectively.

### Associations with ER-specific breast cancer risk

The strongest PRS associations with ER-negative disease were observed for PRS_ER-_ for both *BRCA1* (per SD HR = 1.23, 95% CI = 1.18–1.28, *P* = 2×10^−27^) and *BRCA2* (HR = 1.31, 95% CI = 1.21–1.43, *P* = 1×10^−10^) carriers (Table 1). The PRS_BC_ and PRS_ER+_ showed the strongest associations with ER-positive disease for *BRCA1* and *BRCA2* carriers with similar HR estimates for PRS_BC_ and PRS_ER+_ (Table 1). The associations remained similar after adjusting for family history of breast cancer (Table 1).

### Associations with epithelial ovarian cancer risk

The 30-SNP PRS_EOC_ was strongly associated with EOC risk for *BRCA1* (per SD HR = 1.31, 95% CI = 1.24–1.39, *P* = 1×10^−21^) and *BRCA2* (per SD HR = 1.43, 95% CI = 1.29–1.59, *P* = 2×10^−11^) carriers (Table 1). The 22-SNP PRS_HGS_, based only on SNPs showing associations with high-grade serous EOC, showed similar associations (Table 1, *BRCA1* HR = 1.32, 95% CI = 1.25–1.40, *P* = 3×10^−22^; *BRCA2* HR = 1.44, 95% CI = 1.30–1.60, *P* = 4×10^−12^). Adjusting for family history of ovarian cancer yielded similar associations to unadjusted models (Table 1).

PRS_HGS_ was used for downstream analyses for *BRCA1* and *BRCA2* carriers. There was no evidence of heterogeneity in the PRS_HGS_ associations across countries (Fig. S3: *BRCA1* P_LRT_ = 0.08; *BRCA2* P_LRT_ = 0.97). For both *BRCA1* and *BRCA2* carriers the estimated HRs by PRS percentile categories (Table 2) were consistent with those expected under the theoretical population-based PRS distributions (Fig. 1c, d). There was no evidence that the PRS_HGS_ association with EOC risk varied by age (*BRCA1* *P* = 0.35; *BRCA2* *P* = 0.14). The associations between PRS_HGS_ and EOC risk varied by *BRCA1* variant location (P_LRT_ = 8.7×10^−3^), with a larger HR for variants in the central region of *BRCA1* (central region HR = 1.50, 95% CI = 1.35–1.66; 5’ to c.2281 region HR = 1.30, 95% CI = 1.18–1.42; c.4072 to 3’ region HR = 1.21, 95% CI = 1.10–1.33). There was little evidence to support differences in the associations by *BRCA2* variant location (Table 2). There was no evidence of differences in the associations by the *BRCA1* variant predicted effect on protein expression (P_difference_ = 0.85).

The C-statistics for PRS_HGS_ were estimated to be 0.604 (95% CI = 0.582–0.626) for *BRCA1* and 0.667 (95% CI = 0.636–0.699) for *BRCA2* carriers.

### Prospective cohort associations

The breast cancer prospective cohort included 2088 *BRCA1* carriers with 297 incident cases and 1757 *BRCA2* carriers with 215 incident cases (Table S4). The PRS_ER-_ was associated with breast cancer risk for *BRCA1* carriers (per SD HR = 1.28, 95% CI = 1.14–1.44, *P* = 4.4×10^−5^). For *BRCA2* carriers, PRS_BC_ was associated with breast cancer risk with a per SD HR = 1.36 (95% CI = 1.17–1.57, *P* = 4.3×10^−5^) (Table 3).

**Table 3:** Associations of the best performing PRS in the prospective cohort of *BRCA1* and *BRCA2* carriers.

Outcome		PRS	Number of women at risk	Incident cancers	HR (95% CI)	*P*
Breast cancer	*BRCA1* carriers	ER-	2088	297	1.28 (1.14–1.44)	4.44×10^−5^
	*BRCA2* carriers	BC	1757	215	1.36 (1.17–1.57)	4.26×10^−5^
Ovarian cancer	*BRCA1* carriers	HGS	3152	108	1.28 (1.06–1.55)	1.08×10^−2^
	*BRCA2* carriers	HGS	2495	56	1.45 (1.13–1.86)	3.29×10^−3^

Number of women at risk is the number of pathogenic variant carriers unaffected at baseline. Incident cancers is the number of women who developed breast/ovarian cancer during the follow-up period.

*BC* breast cancer, *CI* confidence interval, *ER-* estrogen receptor negative, *HGS* high-grade serous, *HR* hazard ratio, *PRS* polygenic risk score.

The ovarian cancer prospective cohort comprised 3152 *BRCA1* carriers with 108 incident cases and 2495 *BRCA2* carriers with 56 incident cases (Table S4). The PRS_HGS_ was associated with EOC risk for both *BRCA1* (HR = 1.28, 95% CI = 1.06–1.55, *P* = 0.011) and *BRCA2* (HR = 1.45, 95% CI = 1.13–1.86, *P* = 0.003) carriers (Table 3).

### Absolute risks of cancer by PRS percentiles

**Fig. 2:**
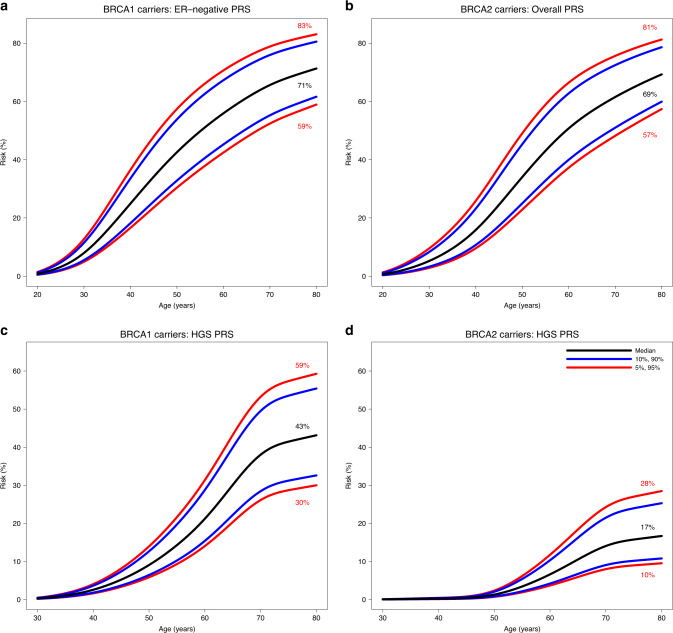
Predicted absolute risks of developing breast and ovarian cancer by polygenic risk score (PRS) percentile. Risks were calculated assuming the retrospective cohort hazard ratio (HR) estimates (Tables 1, 2). (**a**) Predicted absolute risks of developing breast cancer for *BRCA1* carriers by percentiles of the PRS_ER-_. (**b**) Predicted absolute risks of developing breast cancer for *BRCA2* carriers by percentiles of the PRS_BC_. (**c**) Predicted absolute risks of developing ovarian cancer for *BRCA1* carriers by the percentiles of the PRS_HGS_. (**d**) Predicted absolute risks of developing ovarian cancer for *BRCA2* carriers by percentiles of the PRS_HGS_. *ER* estrogen receptor, *HGS* high-grade serous.

We estimated age-specific and 10-year absolute risks of developing breast and ovarian cancers across different PRS percentiles (Figs. 2 and S4). *BRCA1* carriers at the 5th and 95th percentiles of the PRS_ER-_ distribution were predicted to have breast cancer risks to age 80 years of 59% and 83%, respectively. The corresponding risks for *BRCA2* carriers based on PRS_BC_ were 57% and 81%. Although PRS associations were not altered by family history adjustment in the models, and there was no significant evidence of interaction between PRS and variant location, both of these factors remain significant predictors of breast cancer risk (in addition to PRS). Therefore, family history and variant location can be considered jointly with the PRS to predict cancer risks for *BRCA1*/*2* carriers (Figs. S5–S9). For example, breast cancer risk to age 80 years for *BRCA2* carriers with no family history at the 5th and 95th percentiles of the PRS were predicted to be 43% and 67%, respectively, compared with 62% and 85% for those with a family history. The risks of developing ovarian cancer by age 80 years were 30% and 59% for *BRCA1* carriers at the 5th and 95th percentiles of the PRS_HGS_ distribution. The corresponding risks for *BRCA2* carriers were 10% and 28%, respectively.

## DISCUSSION

We investigated the associations between a recently reported PRS for breast cancer, based on 313 SNPs, and a PRS for EOC, based on 30 SNPs, with cancer risks for *BRCA1* and *BRCA2* carriers. The associations were evaluated in a large retrospective cohort and separately in a prospective cohort of *BRCA1*/*2* carriers.

The results demonstrate that the PRS developed using population-based data are also associated with breast and ovarian cancer risk for women with *BRCA1*/*2* pathogenic variants. The PRS developed for predicting ER-negative breast cancer showed the strongest association with breast cancer risk for *BRCA1* carriers, while for *BRCA2* carriers the PRS developed for predicting overall breast cancer risk performed best. The associations were unchanged after adjusting for cancer family history and were similar between the retrospective and prospective studies. There was evidence that the magnitude of the PRS associations decreased with increasing age for *BRCA1* and *BRCA2* carriers. There was evidence for differences in associations by the predicted effects of variants on protein stability/expression, with the breast cancer PRS having a larger effect for carriers of variants predicted to yield a stable protein. For ovarian cancer, the PRS developed for predicting overall or HGS EOC demonstrated similar evidence of association with EOC risk, for both *BRCA1* and *BRCA2* carriers. The results are consistent with findings from a previous CIMBA study, based on fewer samples and fewer SNPs, which demonstrated that PRS can lead to large differences in absolute risks of developing breast and ovarian cancers for female *BRCA1*/*2* carriers.^10^

The estimated HR associations for the PRS with breast cancer risk from this study were smaller than the estimated ORs from the population-based study in which they were derived.^12^ This difference is unlikely to be an overestimation of the ORs in the general population (“winner’s curse”^26^), because the effect sizes were estimated in prospective studies that were independent of the data used in their development.^12,27^ Adjustment for family history, a potential confounder in this study, did not influence the associations. Therefore, these most likely represent real differences, in which PRS modify breast cancer risk for *BRCA1*/*2* carriers to a smaller relative extent than the general population. This meaningful attenuation must be considered when using population-based PRS to predict breast cancer risk for *BRCA1*/*2* carriers and should be incorporated into breast cancer risk prediction models.^28^

The departure from the multiplicative model for the joint effects of PRS (or some subset of SNPs) and *BRCA1*/*2* pathogenic variants might simply reflect the high absolute risks for *BRCA1*/*2* carriers. That is, women with the highest polygenic risk are likely to develop breast cancer at a young age, so that the relative risk associated with the PRS will diminish with age. It is interesting that the decreasing age effect appeared stronger for carriers than the general population, while the relative risk below age 50 years was more comparable with that seen in the general population.^12^ We found that the breast cancer HRs were significantly elevated for carriers of variants that are predicted to generate a stable mutant protein (class II variants). These elevated HRs were similar to the corresponding ORs for association between the PRS and ER-negative (OR = 1.47) and ER-positive (OR = 1.74) breast cancer reported in the general population.^12^ The vast majority of individuals in the general population would be expected to be noncarriers with intact BRCA1/2 protein expression in at-risk tissues, so this observation suggests that some SNPs in the PRS may exert their effect on proteins that interact with stable wildtype or mutant BRCA1 or BRCA2 protein.

We used the ER-specific PRS to assess associations with ER-positive and ER-negative breast cancer for *BRCA1*/*2* carriers. As expected, the PRS developed for ER-positive breast cancer in the general population was the most predictive of ER-positive breast cancer risk for both *BRCA1* and *BRCA2* carriers, and the PRS developed for ER-negative breast cancer was the most predictive of ER-negative breast cancer for both *BRCA1* and *BRCA2* carriers, in line with known differences in ER expression between *BRCA1-* and *BRCA2-*related tumors.^29,30^ These results suggest that further risk prediction improvements can be achieved by estimating the risk of developing ER-specific breast cancer for *BRCA1*/*2* carriers.

Unlike the breast cancer PRS, no systematic evaluation of EOC PRS has been reported in the general population. We therefore included only SNPs identified through GWAS for EOC and its histotypes, using the reported effect sizes as PRS weights. We found that a PRS constructed on the basis of the associations between SNPs and HGS EOC was the most predictive for both *BRCA1* and *BRCA2* carriers, in line with the fact that the majority of tumors in both *BRCA1* and *BRCA2* carriers are HGS.^15^ The estimated HR for PRS_HGS_ was larger for *BRCA2* carriers compared with the *BRCA1* carrier HR estimate. This pattern had been observed previously, based on a smaller sample size and fewer SNPs, but the difference between the HRs observed here is smaller than that reported previously.^10^

Predicted absolute risks for *BRCA1* carriers at the 5th and 95th PRS percentiles at age 50 years varied from 31% to 58% for breast, and from 5% to 13% for ovarian cancer. By age 80 years, they varied from 59% to 83% for breast and from 30% to 59% for ovarian cancer. The corresponding absolute risks for *BRCA2* carriers by age 50 years ranged from 23% to 49% and by age 80 years from 57% to 81% for breast cancer. The ovarian cancer risks by age 80 years varied from 10% to 28%. We also observed differences in the 10-year age-specific risks of cancer for different PRS distribution percentiles (Fig. S4). For example, the estimated 10-year risk of developing breast cancer at age 40 years was 17% and 34% for *BRCA1* carriers at the 5th and 95th percentiles of the PRS for ER-negative breast cancer, respectively. We found no significant attenuation of the PRS associations when adjusting for family history, and no evidence of interaction between PRS and pathogenic variant location. However, family history and variant location are both associated with cancer risk for *BRCA1*/*2* carriers.^2,20–22^ Taken together, the results suggest that when family history and PRS are considered jointly, or when variant location and PRS are considered jointly, both factors influence the risk of developing breast cancer for *BRCA1*/*2* carriers. As a consequence, the differences in absolute risk become larger when the PRS is considered together with family history or variant location (Figs. S5–S9) and demonstrate that the PRS should be considered in combination with other risk factors to provide comprehensive cancer risks for *BRCA1*/*2* carriers.

Strengths of this study include the large cohort sample sizes of *BRCA1*/*2* carriers and use of independent prospective cohort data to validate PRS associations with cancer risks. The similarity in association estimates between the retrospective and prospective analyses suggests that retrospective estimates have not been strongly influenced by potential biases (e.g., survival bias). As the PRS analyzed in this study were originally developed and validated in population-based studies, the associations reported here represent independent evaluations of the PRS in *BRCA1*/*2* carriers. The analyses were also adjusted for cancer family history, hence associations are unlikely to be biased due to confounding.

Limitations of this study include the fact that tumor ER status information was missing on a substantial proportion of the study population. Therefore, we were unable to assess associations with ER-specific breast cancer in the entire sample of *BRCA1*/*2* carriers. The use of PRS developed in the general population means that if there are *BRCA1*- or *BRCA2*-specific modifier SNPs,^4,5^ these may not have been included in the PRS. Therefore, alternative approaches should also investigate developing PRS using data directly from *BRCA1* and *BRCA2* carriers, although much larger sample sizes will be required. We did not present confidence intervals for the predicted PRS-specific absolute risks of breast or ovarian cancer, and the absolute PRS-specific risks by variant location and family history. These predictions critically depend on external cancer incidence estimates for *BRCA1*/*2* pathogenic variant carriers,^2^ which themselves are uncertain and therefore should only be used as a general guide. Future studies should aim to factor in uncertainty in the predicted risks based on all parameters. In addition, the PRS-specific absolute cancer risks overall and by family history or pathogenic variant location should be validated in much larger prospective studies of unaffected carriers. Finally, the present analyses were limited to carriers of European ancestry. Hence the results presented may not be applicable to *BRCA1*/*2* carriers of Asian, African, and other non-European ancestries.

PRS are now being used in risk-stratified screening trials and other implementation studies in the general population.^31^ They are commercially available and have been incorporated in comprehensive cancer risk prediction models.^28,32^ The findings of this study indicate that these PRS, in combination with established risk modifiers (e.g. family history and pathogenic variant characteristics) can be used to provide more personalized cancer risk predictions for carriers, which may assist clinical management decisions. It is therefore important to undertake relevant implementation studies to determine the optimal way of incorporating these PRS into genetic counseling and risk management, and to assess whether PRS on their own or in combination with other risk factors influence the short- or long-term clinical management decisions that female *BRCA1*/*2* carriers make. Furthermore, the available risk models incorporating the effects of *BRCA1*/*2* pathogenic variants^28,32^ and PRS should be validated in large prospective studies of carriers.

## Supplementary information

Supplementary Material

Supplementary Information
