# Supplementary material for: Polygenic risk scores and breast and epithelial ovarian cancer risks for carriers of BRCA1 and BRCA2 pathogenic variants
Source: Genet Med. 2020 Jul 15;22(10):1653–66. doi: 10.1038/s41436-020-0862-x (PMC7521995; doi:10.1038/s41436-020-0862-x)
Supplement: Supplementary file 1 — Supplementary Material [file 41436_2020_862_MOESM1_ESM.pdf]

SUPPLEMENTARY MATERIAL

Ethics Statement

All study participants provided written informed consent and participated in research or clinical studies at the host institute under ethically approved protocols. The studies and their approving institutes are: Australian site of the Breast Cancer Family Registry (BCFR-AU) - The University of Melbourne Health Sciences Human Ethics Sub-Committee; Northern California site of the Breast Cancer Family Registry (BCFR-NC) - Northern California Cancer Center Institutional Review Board; New York site of the Breast Cancer Family Registry (BCFR-NY) - Columbia University Medical Center Institutional Review Board; Ontario site of the Breast Cancer Family Registry (BCFR-ON) - Mount Sinai Hospital Research Ethics Board; Philadelphia site of the Breast Cancer Family Registry (BCFR-PA) - Institutional Review Board Fox Chase Cancer Center; Utah site of the Breast Cancer Family Registry (BCFR-UT) - Institutional Review Board University of Utah; Baltic Familial Breast and Ovarian Cancer Consortium (BFBOCC) - Centrālā medicīnas ētikas Komiteja; Lietuvos Bioetikos Komitetas; BRCA-gene mutations and breast cancer in South African women (BMBSA) - University of Pretoria and Pretoria Academic Hospitals Ethics Committee; Beckman Research Institute of the City of Hope (BRICOH) - City of Hope Medical Center Institutional Review Board; Copenhagen Breast Cancer Study (CBCS) - De Videnskabsetiske Komiteer I Region Hovedsladen; Spanish National Cancer Centre (CNIO) - Instituto de Salud Carlos III Comité de Bioética y Bienestar Animal; City of Hope Cancer Center (COH) - City of Hope Institutional Review Board; CONsorzio Studi ITaliani sui Tumori Ereditari Alla Mammella (CONSIT TEAM) - Comitato Etico Indipendente della Fondazione IRCCS "Istituto Nazionale dei Tumori"; National Centre for Scientific Research Demokritos (DEMOKRITOS) - Bioethics committee of NCSR ‘‘Demokritos’’, 240/EHΔ/11.3; National Centre for Scientific Research Demokritos (DEMOKRITOS) - Papageorgiou Hospital Ethics Committee; Dana Farber Cancer Institute (DFCI) - Dana Farber Cancer Institute Institutional Review Board; Deutsches Krebsforschungszentrum (DKFZ) - Ethik-Kommission des

Klinikums der Universität; Deutsches Krebsforschungszentrum (DKFZ) - Hospital Universitario de San Ignacio Comité de Investigaciones y Etica; Deutsches Krebsforschungszentrum (DKFZ) - Shaukat Khanum Memorial Cancer Hospital and Research Centre Institutional Review Board; Epidemiological study of BRCA1 and BRCA2 mutation carriers (EMBRACE) - Anglia & Oxford MREC; Fox Chase Cancer Center (FCCC) - Institutional Review Board Fox Chase Cancer Center; Fundación Pública Galega de Medicina Xenómica - Comite Autonomico de Etica da Investigacion de Galicia; German Consortium of Hereditary Breast and Ovarian Cancer (GC-HBOC) - Ethik-Kommission der Medizinischen Fakultät der Universät zu Köln; Genetic Modifiers of cancer risk in BRCA1/2 mutation carriers (GEMO) - Comité consultatif sur le traitement de l'information en matière de recherche dans le domaine de la santé; Georgetown University (GEORGETOWN) - MedStar Research Institute - Georgetown University Oncology Institutional Review Board; Ghent University Hospital (G-FAST) - Universitair Ziekenhuis Gent - Ethics Committee; Hospital Clinico San Carlos (HCSC) - Comité Ético de Investigación Clínia Hospital Clínico San Carlos; Helsinki Breast Cancer Study (HEBCS) - Helsingin ja uudenmaan sairaanhoitopiiri (Helsinki University Central Hospital ethics committee); HEreditary Breast and Ovarian study Netherlands (HEBON) - Protocol Toetsingscommissie van het Nederlands Kanker Instituut/Antoni van Leeuwenhoek Ziekenhuis; Molecular Genetic Studies of Breast- and Ovarian Cancer in Hungary (HUNBOCS) - Institutional Review Board of the Hungarian National Institute of Oncology; University Hospital Vall d'Hebron (HVH) - The Hospital Universitario Vall d'Hebron Clinical Research Ethics Committee; Institut Català d'Oncologia (ICO) - Catalan Institute of Oncology Institutional Review Board; International Hereditary Cancer Centre (IHCC) - Komisji Bioetycznej Pomorskiej Akademii Medycznej (Pomeranian Medical University Bioethics Committee); Iceland Landspitali - University Hospital (ILUH) - Vísindasiđanefnd National Boethics Committee; Interdisciplinary Health Research International Team Breast Cancer Susceptibility (INHERIT) - Comité d'éthique de la recherche du Centre Hospitalier Universitaire de Québec; Istituto Oncologico Veneto Hereditary Breast and Ovarian Cancer Study (IOVHBOCS) - Centro Oncologico Regionale

Azienda Ospedale Di Padova Comitato Etico; Portuguese Oncology Institute-Porto Breast Cancer Study - COMISSÃO DE ÉTICA PARA A SAÚDE (CES) ; Kathleen Cuningham Foundation Consortium for Research into Familial Breast Cancer (KCONFAB) - Queensland Institute of Medical Research - Human Research Ethics Committee; Kathleen Cuningham Foundation Consortium for Research into Familial Breast Cancer (KCONFAB) - Peter MacCallum Cancer Centre Ethics Committee; University of Kansas Medical Center(KUMC) - The University of Kansas Medical Center Human Research Protection Program; Mayo Clinic (MAYO) - Mayo Clinic Institutional Review Boards; McGill University (MCGILL) - McGill Faculty of Medicine Institutional Review Board; Modifier Study of Quantitative Effects on Disease (MOD-SQUAD) - Mayo Clinic Institutional Review Boards; Memorial Sloane Kettering Cancer Center (MSKCC) - Human Biospecimen Utilization Committee; Memorial Sloane Kettering Cancer Center (MSKCC) - Memorial Sloan-Kettering Cancer Center IRB; General Hospital Vienna (MUV) - Ethikkommission der Medizinischen Universität Wien; Women’s College Research Institute Hereditary Breast and Ovarian Cancer Study - University of Toronto Health Sciences Review Ethics Board; National Cancer Institute (NCI) - NIH Ethics Office; National Israeli Cancer Control Center (NICCC) - Carmel Medical Center Institutional Review Board (Helsinki Committee); N.N. Petrov Institute of Oncology (NNPIO) - N.N. Petrov Institional Ethical Committee; NorthShore University HealthSystem (NORTHSHORE) - Institutional Review Board of NorthShore University HealthSystem; NRG Oncology (NRG_ONCOLOGY) - Cancer Prevention and Control Protocol Review Committee; Ontario Cancer Genetics Network (OCGN) - University Health Network Research Ethics Board; The Ohio State University Comprehensive Cancer Center (MACBRCA) - The Ohio State University Cancer Institutional Review Board; Odense University Hospital (OUH) - Den Videnskabsetiske Komité for Region Syddanmark; Pisa Breast Cancer Study (PBCS) - Azienda Ospedaliera Pisana Comitato Etico per lo studio del farmaco sull'uomo; Sheba Medical Centre - Chaim Sheba Medical Center IRB; Swedish Breast Cancer Study (SWE-BRCA) - Regionala Etikprövningsnämnden Stockholm; University of Chicago (UCHICAGO) - The University of Chicago Biological Sciences Division

Institutional Review Board (BSD IRB); University of California Los Angeles (UCLA) - UCLA Institutional Review Board (UCLA IRB); University of California San Francisco (UCSF) - Human Research Protection Program Institutional Review Board (IRB); UK and Gilda Radner Familial Ovarian Cancer Registries (UKGRFOCR) - Roswell Park Cancer Institute IRB; UK and Gilda Radner Familial Ovarian Cancer Registries (UKGRFOCR) - Cambridge Local Research Ethics Committee; University of Pennsylvania (UPENN) - University of Pennsylvania Institutional Review Board; Cancer Family Registry University of Pittsburg (UPITT) - University of Pittsburgh Institutional Review Board; University of Texas MD Anderson Cancer Center (UTMDACC) - University of Texas MD Anderson Cancer Center Office of Protocol Research Institutional Review Board; Victorian Familial Cancer Trials Group (VFCTG) - Peter MacCallum Cancer Centre Ethics Committee; Women's Cancer Program at Cedars-Sinai Medical Center (WCP) - (Cedars-Sinai Medical Center) CSMC Institutional Review Board.

Genotyping and SNP imputation

Genotyping was performed on one of two bespoke SNP arrays. The majority of the samples were genotyped using the OncoArray (15,679 *BRCA1* and 10,981 *BRCA2* carriers)[1-3]. The OncoArray is a custom genotyping array comprising approximately 533,000 SNPs, including a GWAS backbone component tagging common SNPs across the genome which accounted for approximately half of the SNPs on the array. The remaining 3,256 (17.2%) *BRCA1* and 1,358 (11.0%) *BRCA2* pathogenic variant carriers were genotyped using the iCOGS array[4,5] which included approximately 210,000 SNPs selected primarily on the basis of evidence of association with breast, ovarian and prostate cancers.

A standard quality control (QC) process was applied for samples genotyped on both arrays, which included assessment of the SNP call rate, allele frequency, genotyping intensity clustering metrics, Hardy-Weinberg equilibrium and SNP concordance in duplicate samples[2]. Two-stage imputation was performed using SHAPEIT software[6] for phasing and IMPUTE2 software[7] for imputation using the 1000 Genomes Project (Phase 3) reference

panel.

SNPs were included in the PRS if they were adequately imputed in the CIMBA data. The imputation accuracy was assessed using the r^2 statistic, based on the “info” statistic produced by the IMPUTE2 software (https://mathgen.stats.ox.ac.uk/impute/impute_v2.html#info_metric_details)[7]. This statistic takes values from 0 (complete uncertainty of imputed genotypes) to 1 (no uncertainty of imputed genotypes). The r^2 values for the SNPs used in the current analyses are listed in Tables S1 and S2 and shown in Figures S1 and S2.The minimum r^2 values among the 313 SNPs in the breast cancer PRS were 0.49 for samples genotyped on iCOGS and 0.90 for samples genotyped on OncoArray. For the 30 SNPs in the ovarian cancer PRS, the minimum r^2 was 0.64 for iCOGS samples and 0.88 for OncoArray samples.

Principal components analysis

To adjust for potential (intra-continental) population stratification in the OncoArray dataset, principal components analysis was performed using data from 33,661 uncorrelated SNPs (which included 2,318 SNPs specifically selected on informativeness for determining continental ancestry) with a MAF of at least 0.05 and maximum correlation of 0.1 in the OncoArray dataset, using purpose-written software (http://ccge.medschl.cam.ac.uk/software/pccalc). A similar approach was used for the iCOGS dataset.

Breast cancer and epithelial ovarian cancer PRS

PRSs were constructed as the weighted sum of alleles for 313 SNPs for breast cancer and 30 SNPs for epithelial ovarian cancer (EOC), thus the PRS for each participant, *i*, was calculated as:

$$PRS_i = \sum_{j=1}^{N} \beta_j g_{ij}$$

where g_{ij} is the genotype or imputed dosage for variant *j* observed for individual *i* and β_j is

weight for the jth SNP.

The weights for the breast cancer PRS were the log Odds Ratio (log-OR) estimates of association used to construct the 313 SNP PRS based on data from the general population and reported by Mavaddat et al[8]. The weights used to construct the PRS for overall breast cancer (denoted as PRS_{BC}), ER-negative breast cancer (PRS_{ER-}) or ER-positive breast cancer (PRS_{ER+}) are shown in Supplementary Table 1).

Two PRS for epithelial ovarian cancer (EOC) were constructed:

1. A PRS for invasive EOC (PRS_{EOC}) based on 30 SNPs which were: (i) associated with EOC; or (ii) identified through pleiotropic GWAS of breast, EOC and prostate cancer[3,9] at genome-wide significance levels in the combined analyses of the three cancers, but also showed consistent associations with EOC in the Phelan et al[3].
2. A PRS for high grade serous (HGS) ovarian cancer. As HGS is the predominant subtype observed in *BRCA1* and *BRCA2* pathogenic variant carriers[10] a 22 SNP high-grade serous EOC PRS (PRS_{HGS}) was constructed. This PRS was restricted to SNPs that exhibited associations at genome-wide significance level ($P<5x10^{-8}$) with any EOC histotype, was nominally associated ($P<0.05$) with HGS EOC, and the direction of the association for HGS EOC was consistent with the EOC association[3].

The SNPs and the corresponding log-OR weights used in the PRS_{EOC} and PRS_{HGS} are shown in Supplementary Table 2.

Calculating the theoretical PRS

The theoretical PRS distribution under a multiplicative model was used in comparisons against the PRS percentile specific association estimates. For the theoretical PRS, the variance attributable to SNP *i* was given by:

$$V_i = (1-p_i)^2E_i^2 + 2p_i(1-p_i)(\beta_i - E_i)^2 + p_i^2(2\beta - E_i)^2$$

where E_i is the expected value of β, given by:

$$E_i = 2p_i(1-p_i)\beta_i + 2p_i^2\beta_i$$

where β_i is the per-allele log-OR and p_i is the allele frequency for SNP *i*[11] and were obtained from the population-data used in the PRS construction for breast and ovarian cancer (Tables S1 and S2)[3,8]. The mean PRS is then given by:

$$\overline{PRS} = \sum_{i=1}^{N} E_i$$

and the theoretical PRS variance is given by:

$$V = \sum_{i=1}^{N} V_i$$

The allele frequencies were obtained from the 1000 Genomes Project European ancestry samples. The theoretical HRs at each percentile were calculated assuming the PRS is normally distributed with mean $\overline{PRS}$ and variance V (i.e. the HRs were log-normally distributed).

Description of statistical models

Weighted cohort analysis

The retrospective cohort association analyses were undertaken using weighted Cox regression models[12]. These analyses accounted for the non-random sampling of *BRCA1* and *BRCA2* carriers with respect to their disease (breast cancer and ovarian cancer) status. In such retrospective studies, affected carriers tend to be oversampled because *BRCA1* and *BRCA2* testing is targeted to affected individuals who may also be diagnosed at an early age. Therefore, the carriers in CIMBA retrospective study do not represent a true cohort of *BRCA1* and *BRCA2* carriers. We have previously shown that under these conditions, standard Cox regression analysis leads to biased estimates of the rate ratios[12,13]. To correct for this bias, we used the weighted cohort approach[12,13]. Briefly, this method involves assigning different weights to cancer cases and unaffected individuals which are age- and gene-specific, such that the weighted observed incidence rates are consistent with established incidence rates for carriers of pathogenic variants in *BRCA1* and *BRCA2*[14]. This

approach has been shown in simulation studies to yield unbiased estimates of association[12,13]. The weighted cohort analysis was carried out in R “survival” library command `coxph(model,robust=TRUE,weights=w)` where w represents the age specific weights.

Model comparisons

Likelihood ratio tests (LRTs) were undertaken to determine whether the models which include interaction terms (age-varying PRS, PRS interaction with gene variant location and PRS interaction with gene variant class) fitted data better than the nested model that did not include the interaction term. Here we considered two models: (i) a model that includes the PRS interaction term, with a corresponding log-likelihood, L_I and the nested model without the interaction term with log-likelihood L_N. Hence, the LRT comparing these models has the form:

$$-2[L_N - L_I] \sim \chi^2_{\Delta d}$$

where Δd denotes degrees of freedom, given by the difference in number of parameters estimated between the two models.

BRCA1 **and** ***BRCA2*** **Cohort Consortium (BBCC) prospective cohort**

The BBCC included data from the International *BRCA1*/*2* Carrier Cohort Study (IBCCS), Breast Cancer Family Registry (BCFR) and the Kathleen Cunningham Foundation Consortium for Research into Familial Breast Cancer (kConFab) IBCCS study participants were recruited between 1997 and 2011 from 18 European cancer genetics centres and Quebec, Canada. Most women were recruited through large national studies in the United Kingdom, Netherlands and France. All centres actively followed participants through questionnaires. Additionally, where possible, passive follow-up by pathology (Denmark and Netherlands), cancer and death registry linkage (Denmark, Netherlands, Sweden and the United Kingdom), and validation of self-reported cancer diagnoses and preventive surgeries

through medical records.

The BCFR is a family cohort recruited from six sites from Australia, Canada and the USA. The families were followed-up regularly by annual contact of probands and systematic 5-year follow-up of families that collected demographic and epidemiological data from all study participants.

The kConFab recruited pathogenic variant carriers from multi-case families that had been ascertained since 1997 by family cancer clinics in Australia and New Zealand. kConFab study participants were independent from BCFR participants from Australia. Study participants were systematically followed by the kConFab Follow-Up Study[15] using mailed questionnaires every three years, with self-reported cancers and prophylactic surgeries confirmed from medical records. BBCC follow-up ended in December 2013[16].

Association analysis in prospective cohorts

To assess associations between the PRS and breast cancer risk, eligibility was restricted to female *BRCA1* and *BRCA2* carriers who at completion of the baseline questionnaire were free of any cancer diagnosis (excluding non-melanoma skin cancer) and had not undergone risk-reducing bilateral mastectomy. Study participants were followed from baseline until the first of: (i) age 80-years; (ii) death; (iii) completion of last follow-up questionnaire or last record linkage, whichever occurred last; (iv) risk-reducing bilateral mastectomy; or (v) diagnosis of any first cancer (apart from non-melanoma skin cancer). Participants diagnosed with a first breast cancer were considered affected.

To assess associations between the PRS and ovarian cancer risk, eligibility was restricted to women who had not been diagnosed with ovarian cancer and had not had RRSO at the time of baseline questionnaire completion. To maximise statistical power, carriers with a prior breast cancer or non-melanoma skin cancer diagnosis were retained for the prospective ovarian cancer analyses, but carriers with prior diagnoses of other cancers were excluded, in line with previous prospective studies of ovarian cancer risk for *BRCA1/2* pathogenic variant carriers[16]. Participants were followed from baseline until the first of: (i)

age 80-years; (ii) death; (iii) completion of last follow-up questionnaire or record linkage, whichever happened last; (iv) bilateral RRSO, or bilateral salpingectomy, or removal of both ovaries for any other reason; or (v) any cancer diagnosis (except breast or non-melanoma skin cancer). Carriers diagnosed with invasive ovarian, fallopian tube, or peritoneal cancer during the follow-up were considered affected.

Associations using the harmonised prospective cohorts were analysed using Cox regression, separately for *BRCA1* and *BRCA2* carriers. Statistical models were stratified by consortia (CIMBA or BBCC), birth cohort, country, and Ashkenazi Jewish ancestry, adjusted for family history of the appropriate cancer in first- and second-degree relatives. Robust variance estimates were calculated considering family membership.

Calculating absolute cancer risks by PRS

Breast cancer absolute risks were calculated by PRS category and also by PRS category in combination with variant locations in *BRCA1* and *BRCA2*, and in combination with family history of breast cancer (absence or presence of family history).

For these calculations we assumed external estimates of overall breast cancer incidence and breast cancer incidence estimates for different pathogenic variant locations and cancer family history status. The external estimates were obtained from previously published prospective penetrance studies in *BRCA1* and *BRCA2* pathogenic variant carriers[16]. For all analyses, to obtain the breast cancer incidences for each PRS percentile category, we constrained the breast cancer incidences over all PRS categories to agree with the prospectively estimated breast cancer incidence rates using the constraining approach described previously[8,16-18]. In this we assume that the breast cancer incidence for someone in PRS category c is given by $\lambda_0(t)\exp(\beta_c)$ where $\lambda_0(t)$ is the baseline incidence (for those in the baseline PRS category) which is unknown, and β_c is the corresponding log-HR of association with breast cancer risk for a carrier in category *c* relative to the baseline category. Given this constraining, it was previously shown[16,17] that $\lambda_0(t)$ is given by:

$$\lambda_0(t) = i(t)\frac{\sum_c \tau_c S_c(t-1)}{\sum_c \tau_c \exp(\beta_c) S_c(t-1)}$$

where $i(t)$ is the assumed external incidence (i.e. the average over all PRS effects), τ_c is the proportion of carriers in PRS category c and $S_c(t)$ is the probability of surviving the disease to age t in PRS category c. $\lambda_0(t)$ can be calculated iteratively assuming $S_c(0)=1$ over the ages t. Once $\lambda_0(t)$ was calculated, the incidence for each PRS category is given by: $\lambda_0(t)\exp(\beta_c)$. This process was carried out assuming the external incidence estimates for overall breast cancer, incidences by pathogenic variant location or by family history separately[16].

Calculating 10-year cancer risks

The 10-year risk of developing breast or ovarian cancer at age t was calculated as the risk difference between ages (t+10) and t, conditional on not developing cancer up to age t. Mathematically this can be written as:

$$R(t)_{10} = \frac{P(t+10) - P(t)}{1 - P(t)}$$

where $R(t)_{10}$ is the 10-year risk and $P(t)$ is the cumulative disease risk at age t and is calculated using the PRS specific incidences calculated in the previous section.

Supplementary Results

Absolute risks by PRS, variant location and family history (results)

Carriers of pathogenic variants in the non-central gene regions had greater risk of developing breast cancer (5th-95th PRS percentiles *BRCA1* 5' end 61%-88%, 3' end 60%-91%; *BRCA2* (narrow) 5' end 62%-87%, 3' end 60%-92%; *BRCA2* (wide) 5' end 67%-91%, 3' end 61%-93%) compared to carriers with variants in the central regions (*BRCA1* 49%-75%; *BRCA2* (narrow) 42%-73%; *BRCA2* (wide) 41%-71%) (Table S5; Figures S5-S7).

Carriers with a family history of breast cancer (at least one affected first or second degree relative) had larger absolute risks of developing breast cancer up to age 80-years (*BRCA1* 65%-88%; *BRCA2* 62%-85%) compared with carriers without a family history of breast cancer (*BRCA1* 46%-71%; *BRCA2* 62%-85%) (Table S5; Figures S8-S9).

Detailed breast and ovarian cancer absolute risks by PRS percentiles (results)

Table S6 illustrates the absolute risks by age 80-years of developing breast cancer and ovarian cancer for pathogenic variant carriers. These absolute risks are presented for the PRS deciles as well as the most extreme first (i.e. 1st and 99th) and fifth (i.e. 5th and 95th) PRS percentiles. The PRS_{ER-} is presented for *BRCA1* carriers with respect to their breast cancer risk, whilst the PRS_{BC} is shown for *BRCA2* carrier breast cancer risk. The PRS_{HGS} is presented for both *BRCA1* and *BRCA2* carriers with respect to ovarian cancer risks.

Statistical software (R and Stata) commands used for statistical analyses

R Cox regression

```
library(survival)
coxph(Surv(CENSORING.AGE, CENSORING.STATUS) ~ strata(STRATA) +
cluster(FAMILY) + BIRTH.COHORT + PRINCIPAL.COMPONENTS +
NORMALISED.PRS, robust = TRUE, weights = WEIGHTS, data=DATA)
```

Stata age-varying PRS analysis

```
stset CENSORINGAGE [pweight = WEIGHTS], id(ID) f(CENSORINGSTATUS)
xi: stcox NORMALISEDPRS i.BIRTHCOHORT PRINCIPALCOMPONENTS*, ///
strata(STRATA) cluster(FAMILY) tvc(NORMALISEDPRS)
// PRINCIPALCOMPONENTS* represents all principal components
```

Stata: C-index for discrimination

```
net from http://www.homepages.ucl.ac.uk/~rmjwiww/stata/epi
net install cindex
* Fit appropriate Cox model and obtain linear predictions
stcox ...
predict LINPRED, xb
set seed 25456
bootstrap c = r(C_adj_correct2), cluster(FAMILY) reps(1000): cindex
LINPRED, ///
strata(STRATA) adj(_IBIRTHCOHORT* PRINCIPALCOMPONENTS*)
// PRINCIPALCOMPONENTS* represents all principal components
// _IBIRTHCOHORT* represents all birth cohorts (created internally
from the "BIRTHCOHORT" variable by Stata after fitting the -stcox-
model)
```

SUPPLEMENTARY TABLE LEGENDS

Table S1

The 313 SNPs used to construct the breast cancer PRS[8]. The same set of 313 SNPs was used to construct the PRS_{ER-} and PRS_{ER+}. The ER-specific PRS used different SNP weights (log-ORs for ER-specific breast cancer) if they had a statistically significant different effect on ER-subtype from a population-based breast cancer case-only analysis.

Table S2

The 30 SNPs used to construct the ovarian cancer PRS. The 22 SNPs used to form the high-grade serous ovarian cancer PRS are highlighted in grey. The high-grade serous specific PRS was limited to SNPs that showed genome-wide statistical significance ($P<5x10^{-8}$) with any of the ten ovarian cancer subtypes, had concordant direction of effects between overall all invasive and high-grade serous disease, and exhibited nominal statistical significance (P<0.05) with high-grade serous ovarian cancer[3]. The “overall” and “high-grade serous” ovarian cancer (per-allele) effect sizes and P-values were taken from[3] and/or[9].

Table S3

Retrospective cohort characteristics for 18,935 *BRCA1* and 12,339 *BRCA2* carriers recruited by the CIMBA. Breast cancer and ovarian cancer refer to the first cancer diagnosis. Censoring ages are reported in years. Pathogenic variant classes: I = unstable or no protein; II = stable mutant protein; III = consequence unknown. Pathogenic variant locations are in base pairs (bp) within the *BRCA1* and *BRCA2* genes. ER-status is oestrogen receptor status of the breast tumour. Cancer family history is reported for the relevant cancer from first and second degree relatives. “Unknown” family history = reported unknown cancer family history, “missing” family history = family history data not collected. IQR = interquartile range; SD = standard deviation.

Table S4

Validation data summary statistics from prospective cohorts (CIMBA and BBCC). Validation data are presented for the breast cancer PRS and ovarian cancer PRS by disease status at censoring. The PRS_{ER-} is reported for *BRCA1* carriers, whilst the PRS_{ER+} is presented for *BRCA2* carriers with respect to the breast cancer data. PRS_{HGS} is shown for both *BRCA1* and *BRCA2* carriers for the ovarian cancer data. The median (IQR) age at start of follow-up, follow-up time and age at cancer diagnosis (years) are displayed. The mean and SD are shown for the appropriate PRS. IQR = interquartile range; N = sample size; SD = standard deviation.

Table S5

Assumed proportions and hazard ratios used to constrain the breast cancer incidences from the external BBCC prospective cohort study for breast cancer family history and gene variant location[16]. The absolute risks of breast cancer at the 5th, 50th and 95th percentiles of the PRS are shown (absolute risk curves are plotted in Figures S5-S9).

Table S6

Absolute breast cancer and ovarian cancer risks by age 80-years for *BRCA1* and *BRCA2* carriers for different PRS percentiles. The reported PRS percentiles are: (i) PRS_{ER-} for *BRCA1* carrier breast cancer; (ii) PRS_{BC} for *BRCA2* carrier breast cancer; (iii) PRS_{HGS} for *BRCA1* carrier ovarian cancer; and (iv) PRS_{HGS} for *BRCA2* carrier ovarian cancer.

SUPPLEMENTARY FIGURE LEGENDS

Figure S1

Histograms of imputation accuracy (r^2 statistics) for the 313 breast cancer PRS SNPs. Imputations were performed separately for genotyping arrays (iCOGS or OncoArray) and separately for *BRCA1* and *BRCA2* carriers. All SNPs were well imputed ($r^2 \geq 0.49$).

Figure S2

Histograms of imputation accuracy (r^2 statistics) for the 30 ovarian cancer PRS SNPs. Imputations were performed separately for genotyping arrays (iCOGS or OncoArray) and separately for *BRCA1* and *BRCA2* carriers. All SNPs were well imputed ($r^2 \geq 0.64$).

Figure S3

Forest plots of country specific PRS hazard ratios estimated using the CIMBA retrospective cohort. These models tested for heterogeneity in PRS effects across countries by fitting a PRS by country interaction term. The baseline country was assumed to be UK/Eire. Heterogeneity was assessed using a likelihood ratio test, comparing the model that included the interaction term to a nested model that did not include the interaction term. (A) PRS_{ER-} was used for *BRCA1* carriers (P_{het}=0.26). (B) PRS_{BC} was used for *BRCA2* carriers (P_{het}=0.58). (C) PRS_{HGS} used for *BRCA1* carriers (P_{het}=0.08). (D) PRS_{HGS} used for *BRCA2* carriers (P_{het}=0.95).

Figure S4

Estimated 10-year risks of developing breast cancer and ovarian cancer by different PRS distribution percentiles.

Figure S5

Predicted age-specific absolute risks of developing breast cancer by PRS_{ER-} percentiles and by *BRCA1* variant location. Risks were calculated assuming the retrospective cohort HR

estimates (Table 2). (A) Predicted absolute risks of developing breast cancer for *BRCA1* carriers with a variant in the 5' to c.2281 region. (B) Predicted absolute risks of developing breast cancer for *BRCA1* carriers with a variant in the c.2282 to c.4071 region. (C) Predicted absolute risks of developing breast cancer for *BRCA1* carriers with a variant in the c.4072 to 3' region.

Figure S6

Predicted age-specific absolute risks of developing breast cancer by PRS_{BC} percentiles and by *BRCA2* variant location (narrow definition). Risks were calculated assuming the retrospective cohort HR estimates (Table 2). (A) Predicted absolute risks of developing breast cancer for *BRCA2* carriers with a variant in the 5' to c.3846 region. (B) Predicted absolute risks of developing breast cancer for *BRCA2* carriers with a variant in the c.3847 to c.6275 region. (C) Predicted absolute risks of developing breast cancer for *BRCA2* carriers with a variant in the c.6276 to 5' region.

Figure S7

Predicted age-specific absolute risks of developing breast cancer by PRS_{BC} percentiles and by *BRCA2* variant location (wide definition). Risks were calculated assuming the retrospective cohort HR estimates (Table 2). (A) Predicted absolute risks of developing breast cancer for *BRCA2* carriers with a variant in the 5' to c.2830 region. (B) Predicted absolute risks of developing breast cancer for *BRCA2* carriers with a variant in the c.2831 to c.6402 region. (C) Predicted absolute risks of developing breast cancer for *BRCA2* carriers with a variant in the c.6403 to 5' region.

Figure S8

BRCA1 carriers*:* Predicted age-specific absolute risks of developing breast cancer by PRS_{ER-} percentiles and by family history (FH) of breast cancer. Risks were calculated assuming the retrospective cohort HR estimates (Table 2). (A) Predicted absolute risks of

developing breast cancer for *BRCA1* carriers with no family history of breast cancer. (B) Predicted absolute risks of developing breast cancer for *BRCA1* carriers with positive family history of breast cancer.

Figure S9

BRCA2 carriers: Predicted age-specific absolute risks of developing breast cancer by PRS_{BC} percentiles and by family history (FH) of breast cancer. Risks were calculated assuming the retrospective cohort HR estimates (Table 2). (A) Predicted absolute risks of developing breast cancer for *BRCA2* carriers with no family history of breast cancer. (B) Predicted absolute risks of developing breast cancer for *BRCA2* carriers with positive family history of breast cancer.

FUNDING AND ACKNOWLEDGEMENTS

Funding

The CIMBA data management and data analysis were supported by Cancer Research – UK grants C12292/A20861, C12292/A11174. GCT and ABS are NHMRC Research Fellows. iCOGS: the European Community's Seventh Framework Programme under grant agreement n° 223175 (HEALTH-F2-2009-223175) (COGS), Cancer Research UK (C1287/A10118, C1287/A 10710, C12292/A11174, C1281/A12014, C5047/A8384, C5047/A15007, C5047/A10692, C8197/A16565), the National Institutes of Health (CA128978) and Post-Cancer GWAS initiative (1U19 CA148537, 1U19 CA148065 and 1U19 CA148112 - the GAME-ON initiative), the Department of Defence (W81XWH-10-1-0341), the Canadian Institutes of Health Research (CIHR) for the CIHR Team in Familial Risks of Breast Cancer (CRN-87521), and the Ministry of Economic Development, Innovation and Export Trade (PSR-SIIRI-701), Komen Foundation for the Cure, the Breast Cancer Research Foundation, and the Ovarian Cancer Research Fund. The PERSPECTIVE and PERSPECTIVE I&I projects were supported by the Government of Canada through Genome Canada and the Canadian Institutes of Health Research, the Ministry of Economy and Innovation through Genome Québec, and The Quebec Breast Cancer Foundation and the Ontario Research Fund.

BCFR: UM1 CA164920 from the National Cancer Institute. The content of this manuscript does not necessarily reflect the views or policies of the National Cancer Institute or any of the collaborating centers in the Breast Cancer Family Registry (BCFR), nor does mention of trade names, commercial products, or organizations imply endorsement by the US Government or the BCFR. BFBOCC: Lithuania (BFBOCC-LT): Research Council of Lithuania grant SEN-18/2015. BIDMC: Breast Cancer Research Foundation. BMBSA: Cancer Association of South Africa (PI Elizabeth J. van Rensburg). CNIO: Spanish Ministry of Health PI16/00440 supported by FEDER funds, the Spanish Instituto de Salud Carlos III (grant PI19/00640) supported by FEDAR funds and the Spanish Research Network on Rare

diseases (CIBERER). COH-CCGCRN: Research reported in this publication was supported by the National Cancer Institute of the National Institutes of Health under grant number R25CA112486, and RC4CA153828 (PI: J. Weitzel) from the National Cancer Institute and the Office of the Director, National Institutes of Health. The content is solely the responsibility of the authors and does not necessarily represent the official views of the National Institutes of Health. CONSIT TEAM: Associazione Italiana Ricerca sul Cancro (AIRC; IG2014 no.15547) to P. Radice. Funds from Italian citizens who allocated the 5x1000 share of their tax payment in support of the Fondazione IRCCS Istituto Nazionale Tumori, according to Italian laws (INT-Institutional strategic projects '5x1000') to S. Manoukian. Associazione Italiana Ricerca sul Cancro (AIRC; IG2015 no.16732) to P. Peterlongo**.** DEMOKRITOS: European Union (European Social Fund – ESF) and Greek national funds through the Operational Program "Education and Lifelong Learning" of the National Strategic Reference Framework (NSRF) - Research Funding Program of the General Secretariat for Research & Technology: SYN11_10_19 NBCA. Investing in knowledge society through the European Social Fund. DFKZ: German Cancer Research Center. EMBRACE: Cancer Research UK Grants C1287/A17523, C1287/A26886 and C1287/A23382. D. Gareth Evans is supported by an NIHR grant to the Biomedical Research Centre, Manchester (NIHR grant IS-BRC-1215-20007). The Investigators at The Institute of Cancer Research and The Royal Marsden NHS Foundation Trust are supported by an NIHR grant to the Biomedical Research Centre at The Institute of Cancer Research and The Royal Marsden NHS Foundation Trust. Ros Eeles and Elizabeth Bancroft are supported by Cancer Research UK Grant C5047/A8385. Ros Eeles is also supported by NIHR support to the Biomedical Research Centre at The Institute of Cancer Research and The Royal Marsden NHS Foundation Trust. FCCC: The University of Kansas Cancer Center (P30 CA168524) and the Kansas Bioscience Authority Eminent Scholar Program. A.K.G. was funded by R0 1CA140323, R01 CA214545, and by the Chancellors Distinguished Chair in Biomedical Sciences Professorship. Ana Vega is supported by the Spanish Health Research Foundation, Instituto de Salud Carlos III (ISCIII) through Research Activity Intensification Program (contract grant numbers: INT15/00070,

INT16/00154, INT17/00133), and through Centro de Investigación Biomédica en Red de Enferemdades Raras CIBERER (ACCI 2016: ER17P1AC7112/2018); Autonomous Government of Galicia (Consolidation and structuring program: IN607B), and by the Fundación Mutua Madrileña (call 2018). GC-HBOC: German Cancer Aid (grant no 110837, Rita K. Schmutzler) and the European Regional Development Fund and Free State of Saxony, Germany (LIFE - Leipzig Research Centre for Civilization Diseases, project numbers 713-241202, 713-241202, 14505/2470, 14575/2470). GEMO: Ligue Nationale Contre le Cancer; the Association “Le cancer du sein, parlons-en!” Award, the Canadian Institutes of Health Research for the "CIHR Team in Familial Risks of Breast Cancer" program and the French National Institute of Cancer (INCa grants 2013-1-BCB-01-ICH-1 and SHS-E-SP 18-015). GEORGETOWN: the Non-Therapeutic Subject Registry Shared Resource at Georgetown University (NIH/NCI grant P30-CA051008), the Fisher Center for Hereditary Cancer and Clinical Genomics Research, and Swing Fore the Cure. G-FAST: Bruce Poppe is a senior clinical investigator of FWO. Mattias Van Heetvelde obtained funding from IWT. HCSC: Spanish Ministry of Health PI15/00059, PI16/01292, and CB-161200301 CIBERONC from ISCIII (Spain), partially supported by European Regional Development FEDER funds. HEBCS: Helsinki University Hospital Research Fund, the Finnish Cancer Society and the Sigrid Juselius Foundation. The HEBON study is supported by the Dutch Cancer Society grants NKI1998-1854, NKI2004-3088, NKI2007-3756, the Netherlands Organisation of Scientific Research grant NWO 91109024, the Pink Ribbon grants 110005 and 2014-187.WO76, the BBMRI grant NWO 184.021.007/CP46 and the Transcan grant JTC 2012 Cancer 12-054. HRBCP: Hong Kong Sanatorium and Hospital, Dr Ellen Li Charitable Foundation, The Kerry Group Kuok Foundation, National Institute of Health1R 03CA130065, and North California Cancer Center. HUNBOCS: Hungarian Research Grants KTIA-OTKA CK-80745 and NKFI_OTKA K-112228. ICO: The authors would like to particularly acknowledge the support of the Asociación Española Contra el Cáncer (AECC), the Instituto de Salud Carlos III (organismo adscrito al Ministerio de Economía y Competitividad) and “Fondo Europeo de Desarrollo Regional (FEDER), una

manera de hacer Europa" (PI10/01422, PI13/00285, PIE13/00022, PI15/00854, PI16/00563 and CIBERONC) and the Institut Català de la Salut and Autonomous Government of Catalonia (2009SGR290, 2014SGR338 and PERIS Project MedPerCan). IHCC: PBZ_KBN_122/P05/2004. ILUH: Icelandic Association "Walking for Breast Cancer Research" and by the Landspitali University Hospital Research Fund. INHERIT: Canadian Institutes of Health Research for the "CIHR Team in Familial Risks of Breast Cancer" program – grant # CRN-87521 and the Ministry of Economic Development, Innovation and Export Trade – grant # PSR-SIIRI-701. IOVHBOCS: Ministero della Salute and "5x1000" Istituto Oncologico Veneto grant. IPOBCS: Liga Portuguesa Contra o Cancro. kConFab: The National Breast Cancer Foundation, and previously by the National Health and Medical Research Council (NHMRC), the Queensland Cancer Fund, the Cancer Councils of New South Wales, Victoria, Tasmania and South Australia, and the Cancer Foundation of Western Australia. KOHBRA: the Korea Health Technology R&D Project through the Korea Health Industry Development Institute (KHIDI), and the National R&D Program for Cancer Control, Ministry of Health & Welfare, Republic of Korea (HI16C1127; 1020350; 1420190). MAYO: NIH grants CA116167, CA192393 and CA176785, an NCI Specialized Program of Research Excellence (SPORE) in Breast Cancer (CA116201), and a grant from the Breast Cancer Research Foundation. MCGILL: Jewish General Hospital Weekend to End Breast Cancer, Quebec Ministry of Economic Development, Innovation and Export Trade. Marc Tischkowitz is supported by the funded by the European Union Seventh Framework Program (2007Y2013)/European Research Council (Grant No. 310018). MODSQUAD: MH CZ - DRO (MMCI, 00209805), MEYS - NPS I - LO1413 to LF, and by Charles University in Prague project UNCE204024 (MZ). MSKCC: the Breast Cancer Research Foundation, the Robert and Kate Niehaus Clinical Cancer Genetics Initiative, the Andrew Sabin Research Fund and a Cancer Center Support Grant/Core Grant (P30 CA008748). NAROD: 1R01 CA149429-01. NCI: the Intramural Research Program of the US National Cancer Institute, NIH, and by support services contracts NO2-CP-11019-50, N02-CP-21013-63 and N02-CP-65504 with Westat, Inc, Rockville, MD. NICCC: Clalit Health Services in Israel, the Israel

Cancer Association and the Breast Cancer Research Foundation (BCRF), NY. NNPIO: the Russian Foundation for Basic Research (grants 17-00-00171, 18-515-45012 and 19-515-25001). NRG Oncology: U10 CA180868, NRG SDMC grant U10 CA180822, NRG Administrative Office and the NRG Tissue Bank (CA 27469), the NRG Statistical and Data Center (CA 37517) and the Intramural Research Program, NCI. OSUCCG: Ohio State University Comprehensive Cancer Center. PBCS: Italian Association of Cancer Research (AIRC) [IG 2013 N.14477] and Tuscany Institute for Tumors (ITT) grant 2014-2015-2016. SEABASS: Ministry of Science, Technology and Innovation, Ministry of Higher Education (UM.C/HlR/MOHE/06) and Cancer Research Initiatives Foundation. SMC: the Israeli Cancer Association. SWE-BRCA: the Swedish Cancer Society. UCHICAGO: NCI Specialized Program of Research Excellence (SPORE) in Breast Cancer (CA125183), R01 CA142996, 1U01CA161032 and by the Ralph and Marion Falk Medical Research Trust, the Entertainment Industry Fund National Women's Cancer Research Alliance and the Breast Cancer research Foundation. OIO is an ACS Clinical Research Professor. UCLA: Jonsson Comprehensive Cancer Center Foundation; Breast Cancer Research Foundation. UCSF: UCSF Cancer Risk Program and Helen Diller Family Comprehensive Cancer Center. UKFOCR: Cancer Research UK. UPENN: National Institutes of Health (NIH) (R01-CA102776 and R01-CA083855; Breast Cancer Research Foundation; Susan G. Komen Foundation for the cure, Basser Research Center for BRCA. UPITT/MWH: Hackers for Hope Pittsburgh. VFCTG: Victorian Cancer Agency, Cancer Australia, National Breast Cancer Foundation. WCP: Dr Karlan is funded by the American Cancer Society Early Detection Professorship (SIOP-06-258-01-COUN) and the National Center for Advancing Translational Sciences (NCATS), Grant UL1TR000124.

This work was supported by the U.S. National Institute of Health (NIH), National Cancer Institute [1RO1CA159868]; as well as by the Breast Cancer Research Foundation; Australia National Health and Medical Research Council [454508, 288704, 145684]; Victorian Health Promotion Foundation; Victorian Breast Cancer Research Consortium; Cancer Australia

[1100868, 809195]; National Breast Cancer Foundation [IF 17]; Queensland Cancer Fund; Cancer Councils of New South Wales, Victoria, Tasmania, and South Australia, and Cancer Foundation of Western Australia. We also thank Heather Thorne, Eveline Niedermayr, Sharon Guo, Stephanie Nesci, Lucy Stanhope, Sarah O'Connor, Sandra Picken, the kConFab research nurses and staff, the heads and staff of the Family Cancer Clinics, and the many families who contribute to kConFab.

KAP is a National Breast Cancer Foundation (Australia) Practitioner Fellow [grant number PRAC-17-004].

M.A. Caligo was supported by Grant 2016 (prog.127/16) from the Fondazione Pisa and by research funding 2017 from the Susan G. Komen Italia onlus.

Acknowledgements

All the families and clinicians who contribute to the studies; Catherine M. Phelan for her contribution to CIMBA until she passed away on 22 September 2017; Sue Healey, in particular taking on the task of pathogenic variant classification with the late Olga Sinilnikova; Maggie Angelakos, Judi Maskiell, Gillian Dite, Helen Tsimiklis; members and participants in the New York site of the Breast Cancer Family Registry; members and participants in the Ontario Familial Breast Cancer Registry; Vilius Rudaitis and Laimonas Griškevičius; Drs Janis Eglitis, Anna Krilova and Aivars Stengrevics; Yuan Chun Ding and Linda Steele for their work in participant enrollment and biospecimen and data management; Alicia Barroso, Rosario Alonso and Guillermo Pita; all the individuals and the researchers who took part in CONSIT TEAM (Consorzio Italiano Tumori Ereditari Alla Mammella), in particular: Daniela Zaffaroni, Irene Feroce, Mariarosaria Calvello, Davide Bondavalli, Aliana Guerrieri Gonzaga, Monica Marabelli, A. Viel, Laura Ottini, Giuseppe Giannini, Gabriele Lorenzo Capone, Liliana Varesco, Viviana Gismondi, Maria Grazia Tibiletti, Ileana Carnevali, Antonella Savarese, Aline Martayan, Stefania Tommasi, Brunella Pilato and the personnel of the Cogentech Cancer Genetic Test Laboratory, Milan, Italy. Ms. JoEllen Weaver and Dr. Betsy Bove; The FPGMX group acknowledges members of the Cancer Genetics group

(IDIS): Ana Blanco, Marta Santamariña and Belinda Rodríguez-Lage; IFE - Leipzig Research Centre for Civilization Diseases (Markus Loeffler, Joachim Thiery, Matthias Nüchter, Ronny Baber); We thank all participants, clinicians, family doctors, researchers, and technicians for their contributions and commitment to the DKFZ study and the collaborating groups in Lahore, Pakistan (Muhammad U. Rashid, Noor Muhammad, Sidra Gull, Seerat Bajwa, Faiz Ali Khan, Humaira Naeemi, Saima Faisal, Asif Loya, Mohammed Aasim Yusuf) and Bogota, Colombia (Diana Torres, Ignacio Briceno, Fabian Gil). Genetic Modifiers of Cancer Risk in BRCA1/2 Mutation Carriers (GEMO) study is a study from the National Cancer Genetics Network UNICANCER Genetic Group, France. We wish to pay a tribute to Olga M. Sinilnikova, who with Dominique Stoppa-Lyonnet initiated and coordinated GEMO until she sadly passed away on the 30th June 2014. The team in Lyon (Olga Sinilnikova, Mélanie Léoné, Laure Barjhoux, Carole Verny-Pierre, Sylvie Mazoyer, Francesca Damiola, Valérie Sornin) managed the GEMO samples until the biological resource centre was transferred to Paris in December 2015 (Noura Mebirouk, Fabienne Lesueur, Dominique Stoppa-Lyonnet). We want to thank all the GEMO collaborating groups for their contribution to this study: Coordinating Centre, Service de Génétique, Institut Curie, Paris, France: Muriel Belotti, Ophélie Bertrand, Anne-Marie Birot, Bruno Buecher, Sandrine Caputo, Anaïs Dupré, Emmanuelle Fourme, Marion Gauthier-Villars, Lisa Golmard, Claude Houdayer, Marine Le Mentec, Virginie Moncoutier, Antoine de Pauw, Claire Saule, Dominique Stoppa-Lyonnet, and Inserm U900, Institut Curie, Paris, France: Fabienne Lesueur, Noura Mebirouk.Contributing Centres : Unité Mixte de Génétique Constitutionnelle des Cancers Fréquents, Hospices Civils de Lyon - Centre Léon Bérard, Lyon, France: Nadia Boutry-Kryza, Alain Calender, Sophie Giraud, Mélanie Léone. Institut Gustave Roussy, Villejuif, France: Brigitte Bressac-de-Paillerets, Olivier Caron, Marine Guillaud-Bataille. Centre Jean Perrin, Clermont–Ferrand, France: Yves-Jean Bignon, Nancy Uhrhammer. Centre Léon Bérard, Lyon, France: Valérie Bonadona, Christine Lasset. Centre François Baclesse, Caen, France: Pascaline Berthet, Laurent Castera, Dominique Vaur. Institut Paoli Calmettes, Marseille, France: Violaine Bourdon, Catherine Noguès, Tetsuro Noguchi, Cornel

Popovici, Audrey Remenieras, Hagay Sobol. CHU Arnaud-de-Villeneuve, Montpellier, France: Isabelle Coupier, Pascal Pujol. Centre Oscar Lambret, Lille, France: Claude Adenis, Aurélie Dumont, Françoise Révillion. Centre Paul Strauss, Strasbourg, France: Danièle Muller. Institut Bergonié, Bordeaux, France: Emmanuelle Barouk-Simonet, Françoise Bonnet, Virginie Bubien, Michel Longy, Nicolas Sevenet, Institut Claudius Regaud, Toulouse, France: Laurence Gladieff, Rosine Guimbaud, Viviane Feillel, Christine Toulas. CHU Grenoble, France: Hélène Dreyfus, Christine Dominique Leroux, Magalie Peysselon, Rebischung. CHU Dijon, France: Amandine Baurand, Geoffrey Bertolone, Fanny Coron, Laurence Faivre, Caroline Jacquot, Sarab Lizard. CHU St-Etienne, France: Caroline Kientz, Marine Lebrun, Fabienne Prieur. Hôtel Dieu Centre Hospitalier, Chambéry, France: Sandra Fert Ferrer. Centre Antoine Lacassagne, Nice, France: Véronique Mari. CHU Limoges, France: Laurence Vénat-Bouvet. CHU Nantes, France: Stéphane Bézieau, Capucine Delnatte. CHU Bretonneau, Tours and Centre Hospitalier de Bourges France: Isabelle Mortemousque. Groupe Hospitalier Pitié-Salpétrière, Paris, France: Chrystelle Colas, Florence Coulet, Florent Soubrier, Mathilde Warcoin. CHU Vandoeuvre-les-Nancy, France: Myriam Bronner, Johanna Sokolowska. CHU Besançon, France: Marie-Agnès Collonge-Rame, Alexandre Damette. CHU Poitiers, Centre Hospitalier d'Angoulême and Centre Hospitalier de Niort, France: Paul Gesta. Centre Hospitalier de La Rochelle: Hakima Lallaoui. CHU Nîmes Carémeau, France: Jean Chiesa. CHI Poissy, France: Denise Molina-Gomes. CHU Angers, France : Olivier Ingster; Ilse Coene en Brecht Crombez; Ilse Coene and Brecht Crombez; Alicia Tosar and Paula Diaque; Drs. Sofia Khan, Taru A. Muranen, Carl Blomqvist, Irja Erkkilä and Virpi Palola; The Hereditary Breast and Ovarian Cancer Research Group Netherlands (HEBON) consists of the following Collaborating Centers: Netherlands Cancer Institute (coordinating center), Amsterdam, NL: M.A. Rookus, F.B.L. Hogervorst, F.E. van Leeuwen, M.A. Adank, M.K. Schmidt, D.J. Jenner; Erasmus Medical Center, Rotterdam, NL: J.M. Collée, A.M.W. van den Ouweland, M.J. Hooning, I.A. Boere; Leiden University Medical Center, NL: C.J. van Asperen, P. Devilee, R.B. van der Luijt, T.C.T.E.F. van Cronenburg; Radboud University Nijmegen Medical Center, NL: M.R.

Wevers, A.R. Mensenkamp; University Medical Center Utrecht, NL: M.G.E.M. Ausems, M.J. Koudijs; Amsterdam Medical Center, NL: T.A.M. van Os; VU University Medical Center, Amsterdam, NL: K. van Engelen, J.J.P. Gille; Maastricht University Medical Center, NL: E.B. Gómez-Garcia, M.J. Blok, M. de Boer; University of Groningen, NL: L.P.V. Berger, A.H. van der Hout, M.J.E. Mourits, G.H. de Bock; The Netherlands Comprehensive Cancer Organisation (IKNL): S. Siesling, J. Verloop; The nationwide network and registry of histo- and cytopathology in The Netherlands (PALGA): E.C. van den Broek. HEBON thanks the study participants and the registration teams of IKNL and PALGA for part of the data collection; Hong Kong Sanatorium and Hospital; the Hungarian Breast and Ovarian Cancer Study Group members (Janos Papp, Aniko Bozsik, Timea Pocza, Zoltan Matrai, Miklos Kasler, Judit Franko, Maria Balogh, Gabriella Domokos, Judit Ferenczi, Department of Molecular Genetics, National Institute of Oncology, Budapest, Hungary) and the clinicians and patients for their contributions to this study; the Oncogenetics Group (VHIO) and the High Risk and Cancer Prevention Unit of the University Hospital Vall d'Hebron, Miguel Servet Progam (CP10/00617), and the Cellex Foundation for providing research facilities and equipment; the ICO Hereditary Cancer Program team led by Dr. Gabriel Capella; the ICO Hereditary Cancer Program team led by Dr. Gabriel Capella; Catarina Santos and Pedro Pinto; members of the Center of Molecular Diagnosis, Oncogenetics Department and Molecular Oncology Research Center of Barretos Cancer Hospital; Heather Thorne, Eveline Niedermayr, all the kConFab research nurses and staff, the heads and staff of the Family Cancer Clinics, and the Clinical Follow Up Study (which has received funding from the NHMRC, the National Breast Cancer Foundation, Cancer Australia, and the National Institute of Health (USA)) for their contributions to this resource, and the many families who contribute to kConFab; the KOBRA Study Group; Csilla Szabo (National Human Genome Research Institute, National Institutes of Health, Bethesda, MD, USA); Eva Machackova (Department of Cancer Epidemiology and Genetics, Masaryk Memorial Cancer Institute and MF MU, Brno, Czech Republic); and Michal Zikan, Petr Pohlreich and Zdenek Kleibl (Oncogynecologic Center and Department of Biochemistry and Experimental Oncology, First

Faculty of Medicine, Charles University, Prague, Czech Republic); Anne Lincoln, Lauren Jacobs; the participants in Hereditary Breast/Ovarian Cancer Study and Breast Imaging Study for their selfless contributions to our research; the NICCC National Familial Cancer Consultation Service team led by Sara Dishon, the lab team led by Dr. Flavio Lejbkowicz, and the research field operations team led by Dr. Mila Pinchev; the investigators of the Australia New Zealand NRG Oncology group; members and participants in the Ontario Cancer Genetics Network; Kevin Sweet, Caroline Craven, Julia Cooper, Amber Aielts, and Michelle O'Conor; Yip Cheng Har, Nur Aishah Mohd Taib, Phuah Sze Yee, Norhashimah Hassan and all the research nurses, research assistants and doctors involved in the MyBrCa Study for assistance in patient recruitment, data collection and sample preparation, Philip Iau, Sng Jen-Hwei and Sharifah Nor Akmal for contributing samples from the Singapore Breast Cancer Study and the HUKM-HKL Study respectively; the Meirav Comprehensive breast cancer center team at the Sheba Medical Center; Christina Selkirk; Håkan Olsson, Helena Jernström, Karin Henriksson, Katja Harbst, Maria Soller, Ulf Kristoffersson; from Gothenburg Sahlgrenska University Hospital: Anna Öfverholm, Margareta Nordling, Per Karlsson, Zakaria Einbeigi; from Stockholm and Karolinska University Hospital: Anna von Wachenfeldt, Annika Lindblom, Brita Arver, Gisela Barbany Bustinza; from Umeå University Hospital: Beatrice Melin, Christina Edwinsdotter Ardnor, Monica Emanuelsson; from Uppsala University: Hans Ehrencrona, Maritta Hellström Pigg, Richard Rosenquist; from Linköping University Hospital: Marie Stenmark-Askmalm, Sigrun Liedgren; Cecilia Zvocec, Qun Niu; Joyce Seldon and Lorna Kwan; Dr. Robert Nussbaum, Beth Crawford, Kate Loranger, Julie Mak, Nicola Stewart, Robin Lee, Amie Blanco and Peggy Conrad and Salina Chan; Simon Gayther, Paul Pharoah, Carole Pye, Patricia Harrington and Eva Wozniak; Geoffrey Lindeman, Marion Harris, Martin Delatycki, Sarah Sawyer, Rebecca Driessen, and Ella Thompson for performing all DNA amplification.

REFERENCES

1. Amos CI, Dennis J, Wang Z, et al. The OncoArray Consortium: A Network for Understanding the Genetic Architecture of Common Cancers. *Cancer Epidemiol Biomarkers Prev.* 2017;26(1):126-135.
2. Milne RL, Kuchenbaecker KB, Michailidou K, et al. Identification of ten variants associated with risk of estrogen-receptor-negative breast cancer. *Nat Genet.* 2017;49(12):1767-1778.
3. Phelan CM, Kuchenbaecker KB, Tyrer JP, et al. Identification of 12 new susceptibility loci for different histotypes of epithelial ovarian cancer. *Nat Genet.* 2017;49(5):680-691.
4. Couch FJ, Wang X, McGuffog L, et al. Genome-wide association study in BRCA1 mutation carriers identifies novel loci associated with breast and ovarian cancer risk. *PLoS Genet.* 2013;9(3):e1003212.
5. Gaudet MM, Kuchenbaecker KB, Vijai J, et al. Identification of a BRCA2-specific modifier locus at 6p24 related to breast cancer risk. *PLoS Genet.* 2013;9(3):e1003173.
6. Delaneau O, Marchini J, Zagury J-F. A linear complexity phasing method for thousands of genomes. *Nat Methods.* 2011;9(2):179-181.
7. Howie BN, Donnelly P, Marchini J. A flexible and accurate genotype imputation method for the next generation of genome-wide association studies. *PLoS Genet.* 2009;5(6):e1000529.
8. Mavaddat N, Michailidou K, Dennis J, et al. Polygenic Risk Scores for Prediction of Breast Cancer and Breast Cancer Subtypes. *Am J Hum Genet.* 2019;104(1):21-34.
9. Kar SP, Beesley J, Amin Al Olama A, et al. Genome-Wide Meta-Analyses of Breast, Ovarian, and Prostate Cancer Association Studies Identify Multiple New Susceptibility Loci Shared by at Least Two Cancer Types. *Cancer Discov.* 2016;6(9):1052-1067.
10. Mavaddat N, Barrowdale D, Andrulis IL, et al. Pathology of breast and ovarian

cancers among BRCA1 and BRCA2 mutation carriers: results from the Consortium of Investigators of Modifiers of BRCA1/2 (CIMBA). *Cancer Epidemiol Biomarkers Prev.* 2012;21(1):134-147.

11. Pharoah PD, Antoniou AC, Easton DF, Ponder BA. Polygenes, risk prediction, and targeted prevention of breast cancer. *N Engl J Med.* 2008;358(26):2796-2803.

12. Antoniou AC, Goldgar DE, Andrieu N, et al. A weighted cohort approach for analysing factors modifying disease risks in carriers of high-risk susceptibility genes. *Genet Epidemiol.* 2005;29(1):1-11.

13. Barnes DR, Lee A, Investigators E, kConFab I, Easton DF, Antoniou AC. Evaluation of association methods for analysing modifiers of disease risk in carriers of high-risk mutations. *Genet Epidemiol.* 2012;36(3):274-291.

14. Antoniou AC, Cunningham AP, Peto J, et al. The BOADICEA model of genetic susceptibility to breast and ovarian cancers: updates and extensions. *Br J Cancer.* 2008;98(8):1457-1466.

15. Phillips K-A, Butow PN, Stewart AE, et al. Predictors of participation in clinical and psychosocial follow-up of the kConFab breast cancer family cohort. *Fam Cancer.* 2005;4(2):105-113.

16. Kuchenbaecker KB, Hopper JL, Barnes DR, et al. Risks of Breast, Ovarian, and Contralateral Breast Cancer for BRCA1 and BRCA2 Mutation Carriers. *JAMA.* 2017;317(23):2402-2416.

17. Antoniou AC, Pharoah PD, McMullan G, Day NE, Ponder BA, Easton D. Evidence for further breast cancer susceptibility genes in addition to BRCA1 and BRCA2 in a population-based study. *Genet Epidemiol.* 2001;21(1):1-18.

18. Kuchenbaecker KB, McGuffog L, Barrowdale D, et al. Evaluation of Polygenic Risk Scores for Breast and Ovarian Cancer Risk Prediction in BRCA1 and BRCA2 Mutation Carriers. *J Natl Cancer Inst.* 2017;109(7).

Figure S1

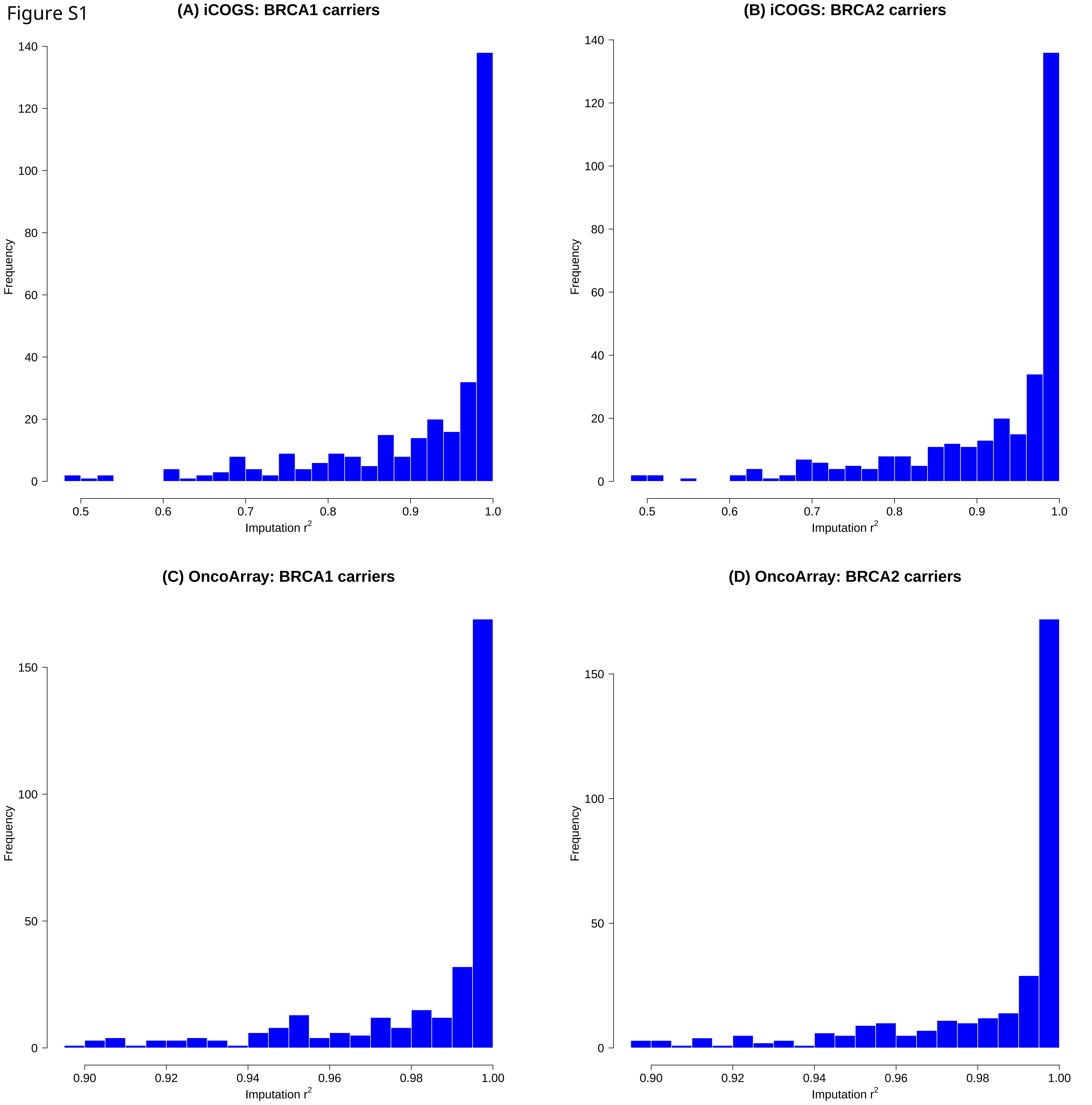

(A) iCOGS: BRCA1 carriers
(B) iCOGS: BRCA2 carriers
(C) OncoArray: BRCA1 carriers
(D) OncoArray: BRCA2 carriers
Frequency
Imputation r2
0
20
40
60
80
100
120
140
150
50
0.5
0.6
0.7
0.8
0.9
1.0
0.90
0.92
0.94
0.96
0.98
1.00

Figure S2

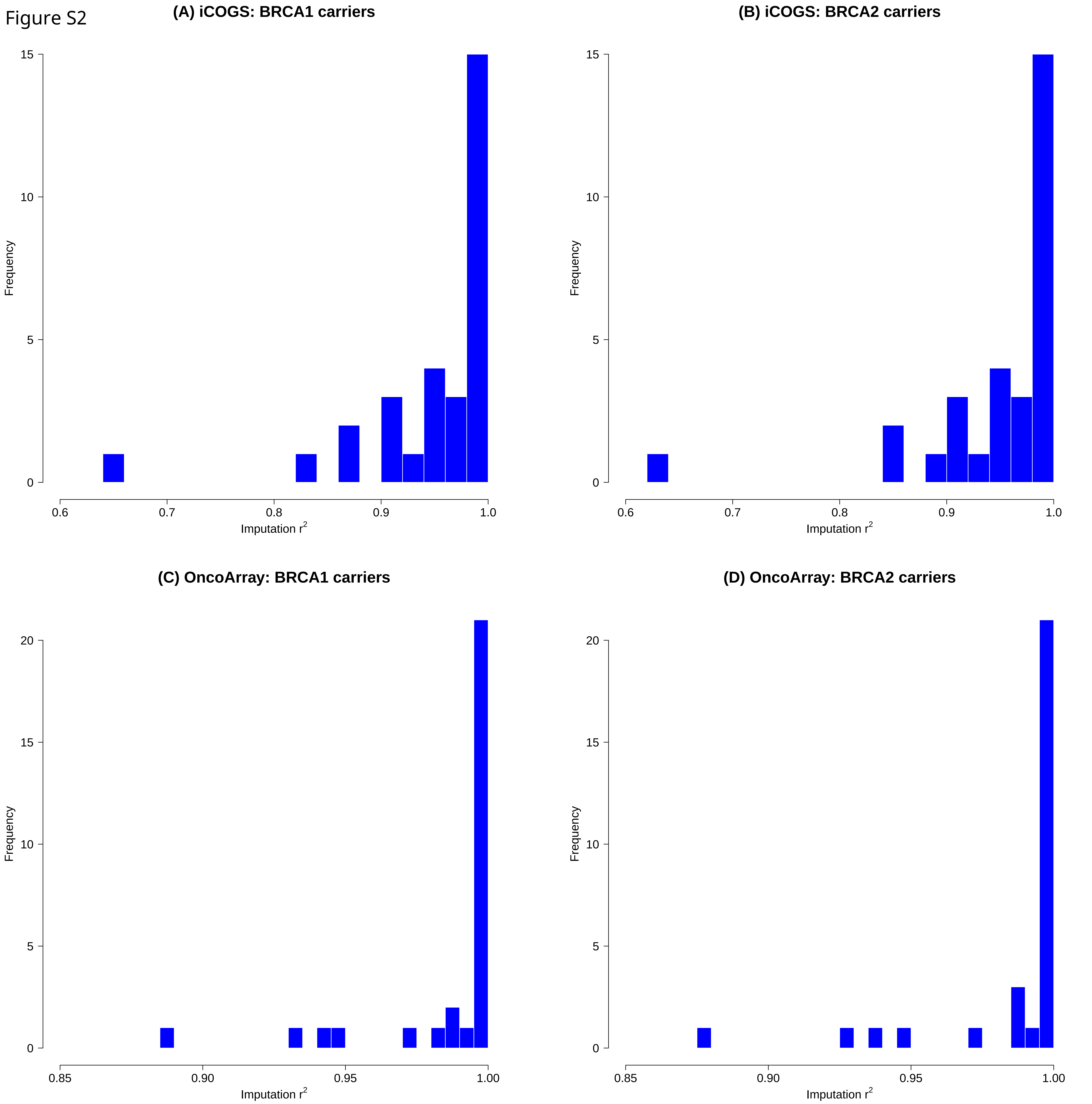
(A) iCOGS: BRCA1 carriers
(B) iCOGS: BRCA2 carriers
(C) OncoArray: BRCA1 carriers
(D) OncoArray: BRCA2 carriers
Frequency
Imputation r2
0
5
10
15
20
0.6
0.7
0.8
0.9
1.0
0.85
0.90
0.95
1.00

Figure S3

(A) BRCA1 carriers: ER−negative PRS

Country	Unaffected	Affected	HR (95% CI)
UK/EIRE	971	822	1.21 (1.11−1.32)
AUSTRALIA	504	525	1.33 (1.17−1.51)
AUSTRIA	277	279	1.29 (1.09−1.53)
BELGIUM	160	163	1.29 (0.98−1.71)
CANADA	363	280	1.22 (1.07−1.39)
CZECH REPUBLIC	68	106	1.59 (1.19−2.12)
DENMARK	556	332	1.25 (1.12−1.41)
FINLAND	71	58	0.97 (0.72−1.29)
FRANCE	666	859	1.27 (1.14−1.41)
GERMANY	749	1316	1.47 (1.33−1.62)
GREECE	97	152	1.47 (1.15−1.88)
HUNGARY	101	179	1.46 (1.14−1.88)
ISRAEL	157	106	1.38 (1.15−1.65)
ITALY	624	673	1.28 (1.16−1.43)
LATVIA	14	30	0.82 (0.52−1.28)
LITHUANIA	135	89	1.15 (0.95−1.41)
NETHERLANDS	663	539	1.37 (1.22−1.55)
POLAND	400	300	1.33 (1.19−1.50)
PORTUGAL	80	38	1.66 (1.31−2.10)
RUSSIA	25	49	1.27 (0.91−1.76)
SOUTH AFRICA	23	38	1.17 (0.74−1.83)
SPAIN	424	399	1.28 (1.12−1.45)
SWEDEN	290	227	1.07 (0.92−1.24)
USA	2044	1914	1.27 (1.19−1.35)
**Main analysis**	**9462**	**9473**	**1.29 (1.25−1.32)**

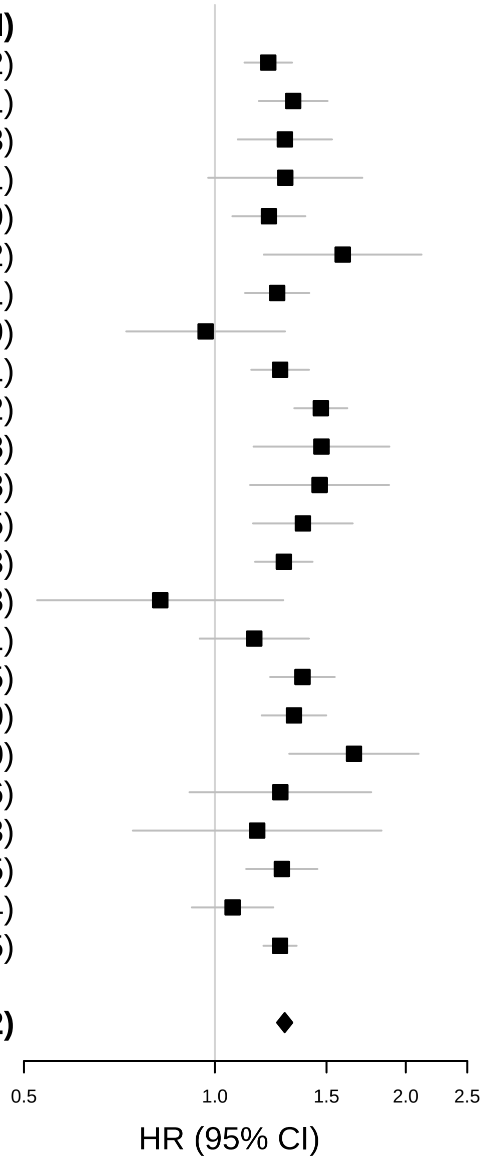

Figure S3

(B) BRCA2 carriers: Overall PRS

Country	Unaffected	Affected	HR (95% CI)
UK/EIRE	897	785	1.42 (1.29−1.55)
AUSTRALIA	433	416	1.32 (1.13−1.54)
AUSTRIA	124	147	1.03 (0.82−1.28)
BELGIUM	87	76	1.34 (1.02−1.74)
CANADA	202	179	1.46 (1.21−1.75)
DENMARK	339	250	1.41 (1.20−1.66)
FINLAND	65	68	1.54 (1.16−2.06)
FRANCE	395	724	1.37 (1.19−1.56)
GERMANY	447	712	1.20 (1.06−1.36)
HUNGARY	48	65	1.44 (0.94−2.19)
ICELAND	28	114	1.01 (0.69−1.47)
ISRAEL	168	106	1.29 (1.03−1.63)
ITALY	246	432	1.31 (1.10−1.55)
NETHERLANDS	469	255	1.30 (1.13−1.49)
PORTUGAL	73	88	1.22 (0.89−1.69)
SOUTH AFRICA	54	92	1.34 (0.93−1.94)
SPAIN	479	508	1.20 (1.06−1.35)
SWEDEN	41	30	1.88 (1.21−2.94)
USA	1397	1248	1.27 (1.18−1.37)
**Main analysis**	**5992**	**6295**	**1.30 (1.26−1.35)**

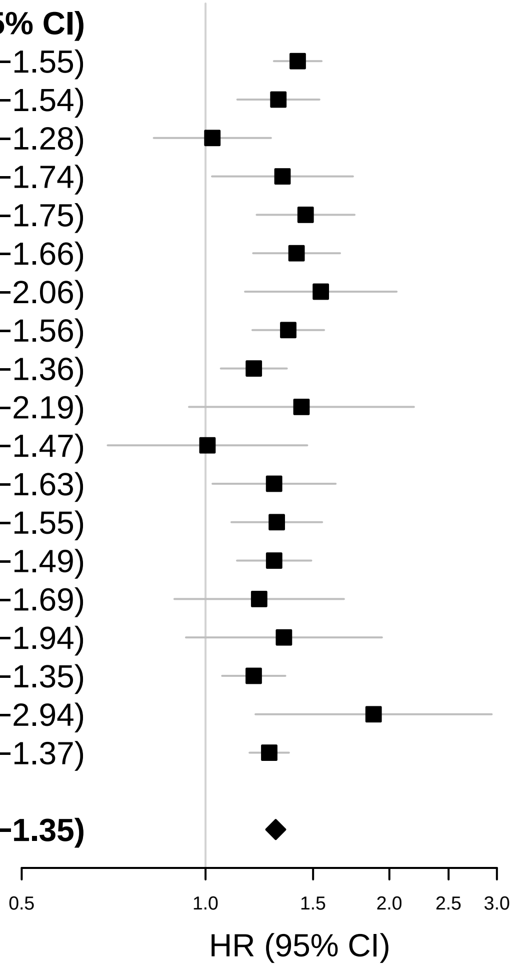

Figure S3

(C) BRCA1 carriers: HGS PRS

Country	Unaffected	Affected	HR (95% CI)
UK/EIRE	1621	172	1.27 (1.06−1.54)
AUSTRALIA	952	77	1.48 (1.16−1.90)
AUSTRIA	509	47	1.63 (1.25−2.13)
BELGIUM	298	25	1.08 (0.68−1.70)
CANADA	574	69	1.26 (0.98−1.61)
CZECH REPUBLIC	142	32	2.36 (1.32−4.24)
DENMARK	770	118	1.52 (1.21−1.92)
FINLAND	114	15	1.25 (0.68−2.31)
FRANCE	1347	178	1.41 (1.15−1.74)
GERMANY	1885	180	1.51 (1.22−1.86)
GREECE	198	51	2.66 (1.95−3.63)
HUNGARY	251	29	0.78 (0.43−1.42)
ISRAEL	225	38	1.05 (0.60−1.84)
ITALY	1045	252	1.26 (0.99−1.60)
LATVIA	32	12	1.73 (0.81−3.71)
LITHUANIA	151	73	1.20 (0.79−1.82)
NETHERLANDS	1112	90	1.27 (1.06−1.52)
POLAND	663	37	1.35 (1.09−1.67)
PORTUGAL	103	15	1.27 (0.75−2.13)
RUSSIA	57	17	1.85 (0.75−4.57)
SOUTH AFRICA	56	5	0.65 (0.31−1.38)
SPAIN	721	102	1.12 (0.87−1.45)
SWEDEN	432	85	1.49 (1.10−2.00)
USA	3609	349	1.24 (1.10−1.40)
**Main analysis**	**16867**	**2068**	**1.33 (1.26−1.41)**

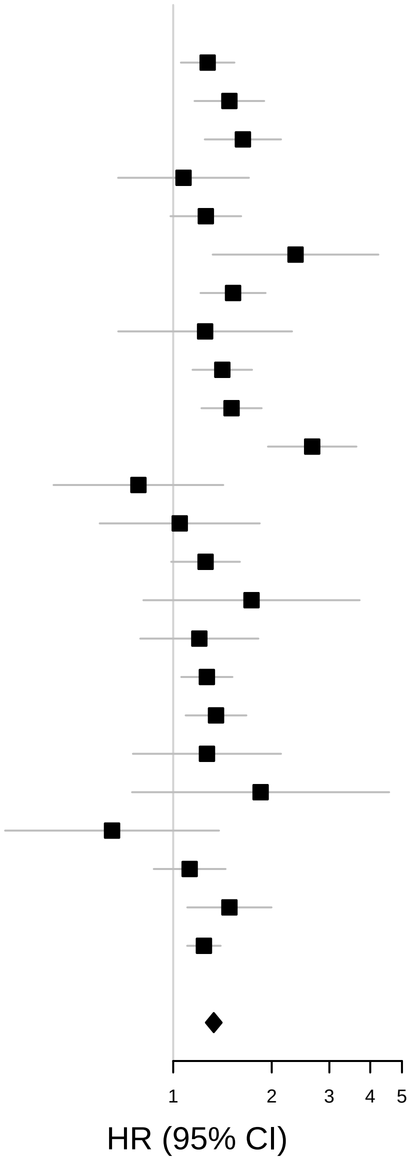

Figure S3

(D) BRCA2 carriers: HGS PRS

Country	Unaffected	Affected	HR (95% CI)
UK/EIRE	1579	103	1.63 (1.27−2.09)
AUSTRALIA	820	29	1.19 (0.89−1.60)
AUSTRIA	262	9	2.25 (1.30−3.87)
BELGIUM/NETHERLANDS	852	35	1.07 (0.77−1.48)
CANADA	355	26	1.52 (0.98−2.37)
DENMARK/ICELAND	687	44	1.47 (1.02−2.13)
FINLAND/LITHUANIA/RUSSIA	145	8	1.00 (0.36−2.78)
FRANCE	1053	66	1.64 (1.09−2.48)
GERMANY	1094	65	1.34 (0.92−1.95)
GREECE/ITALY	653	57	1.29 (0.80−2.07)
HUNGARY	105	8	2.39 (0.86−6.63)
ISRAEL	257	17	0.96 (0.56−1.66)
PORTUGAL/SPAIN	1087	61	1.31 (0.94−1.82)
SOUTH AFRICA	134	12	0.92 (0.14−6.07)
SWEDEN	61	10	1.70 (0.78−3.68)
USA	2477	168	1.52 (1.24−1.86)
**Main analysis**	**16867**	**2068**	**1.42 (1.28−1.57)**

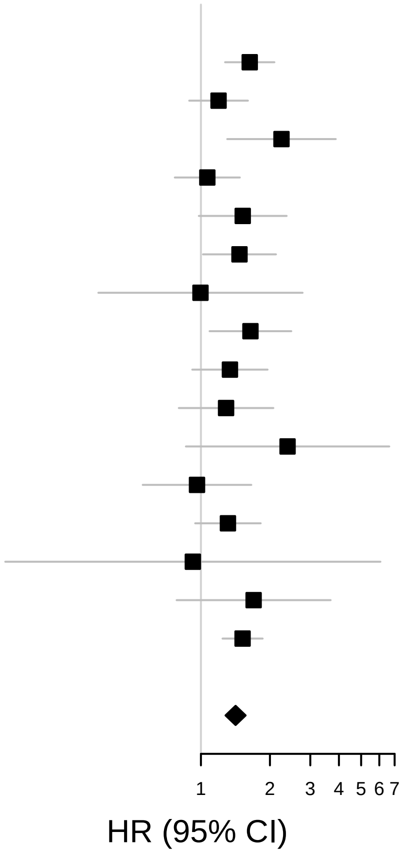

Figure S4

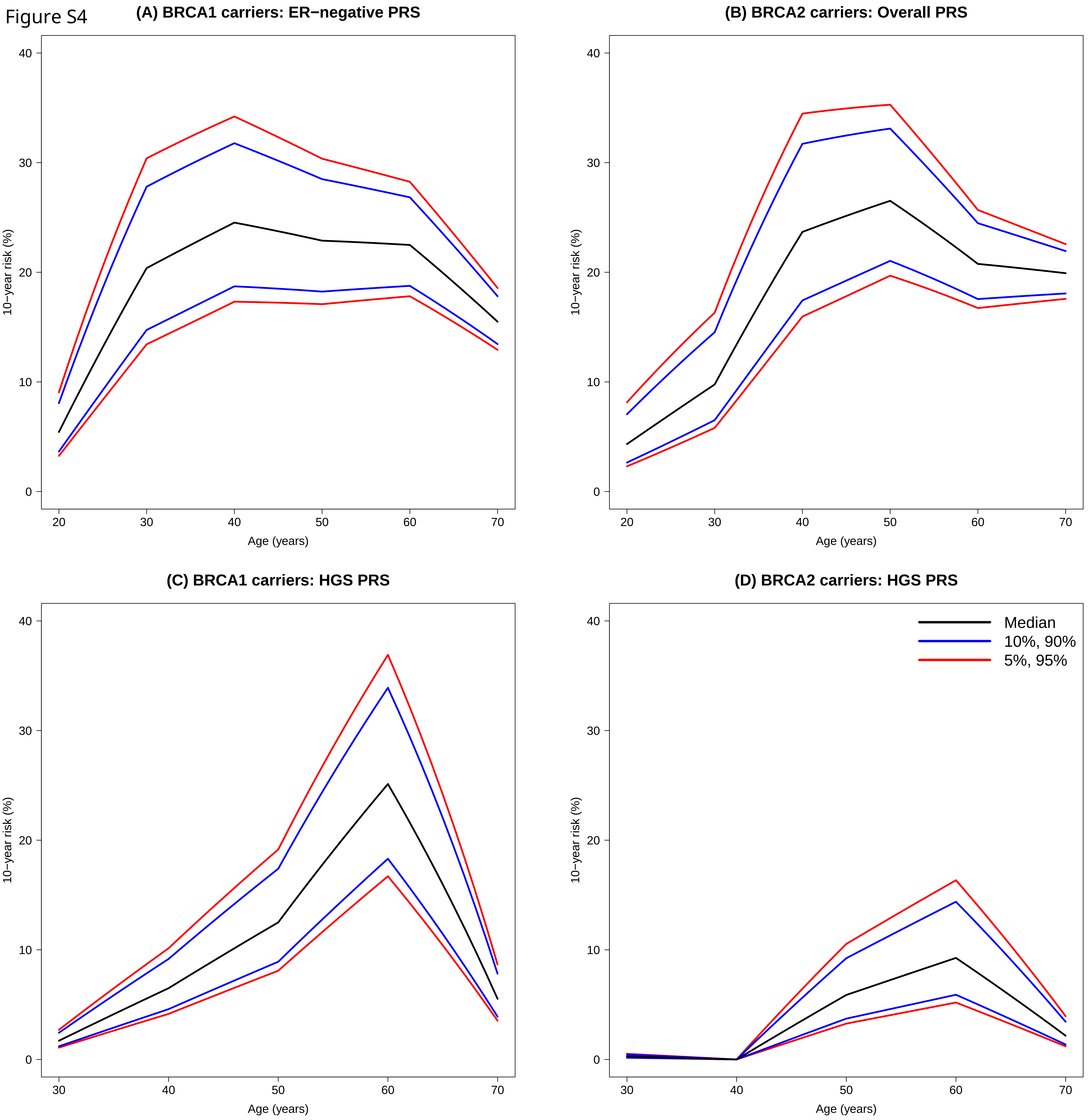
(A) BRCA1 carriers: ER−negative PRS
(B) BRCA2 carriers: Overall PRS
(C) BRCA1 carriers: HGS PRS
(D) BRCA2 carriers: HGS PRS
10−year risk (%)
Age (years)
0
10
20
30
40
20
30
40
50
60
70
Median
10%, 90%
5%, 95%

Figure S5

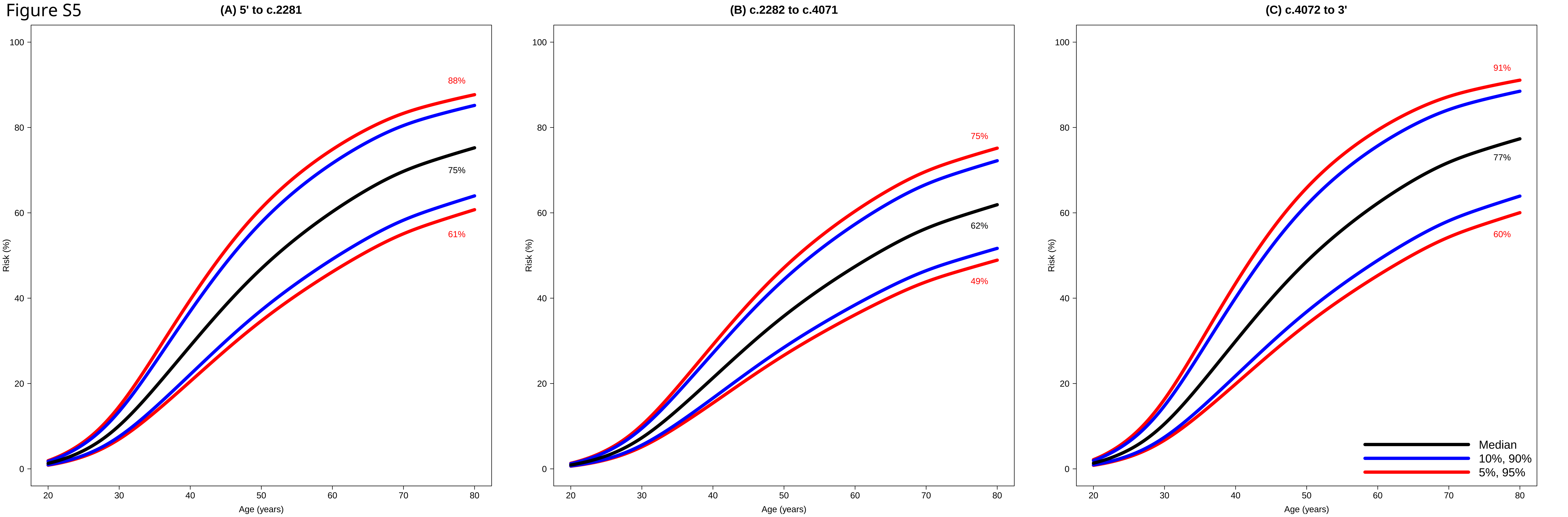
(A) 5' to c.2281
(B) c.2282 to c.4071
(C) c.4072 to 3'
Risk (%)
Age (years)
0
20
40
60
80
100
20
30
40
50
60
70
80
88%
75%
61%
75%
62%
49%
91%
77%
60%
Median
10%, 90%
5%, 95%

Figure S6

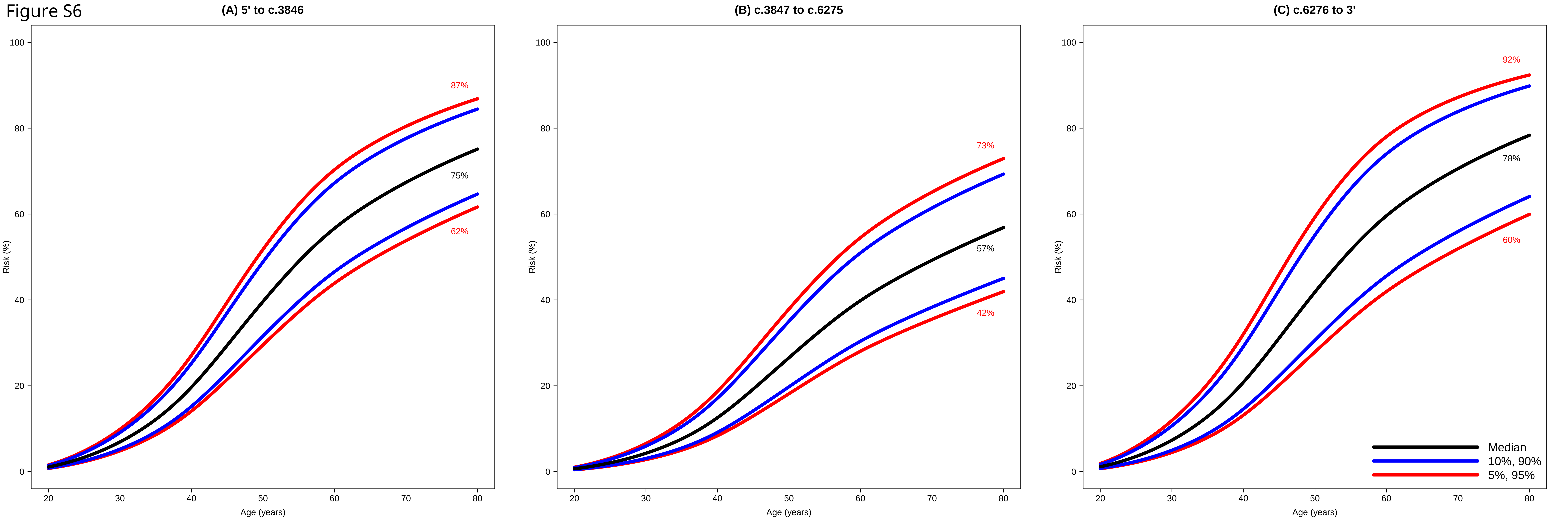

(A) 5' to c.3846
(B) c.3847 to c.6275
(C) c.6276 to 3'
Risk (%)
Age (years)
0
20
40
60
80
100
20
30
40
50
60
70
80
87%
75%
62%
73%
57%
42%
92%
78%
60%
Median
10%, 90%
5%, 95%

Figure S7

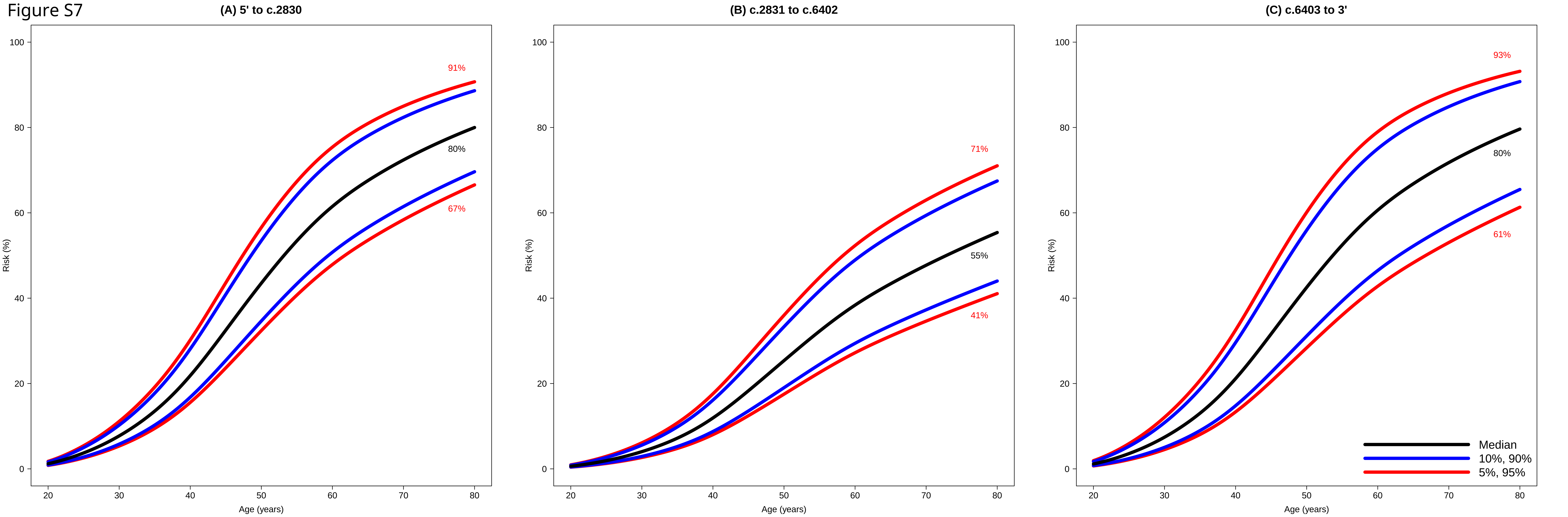

(A) 5' to c.2830
(B) c.2831 to c.6402
(C) c.6403 to 3'
Risk (%)
Age (years)
0
20
40
60
80
100
20
30
40
50
60
70
80
91%
80%
67%
71%
55%
41%
93%
80%
61%
Median
10%, 90%
5%, 95%

Figure S8

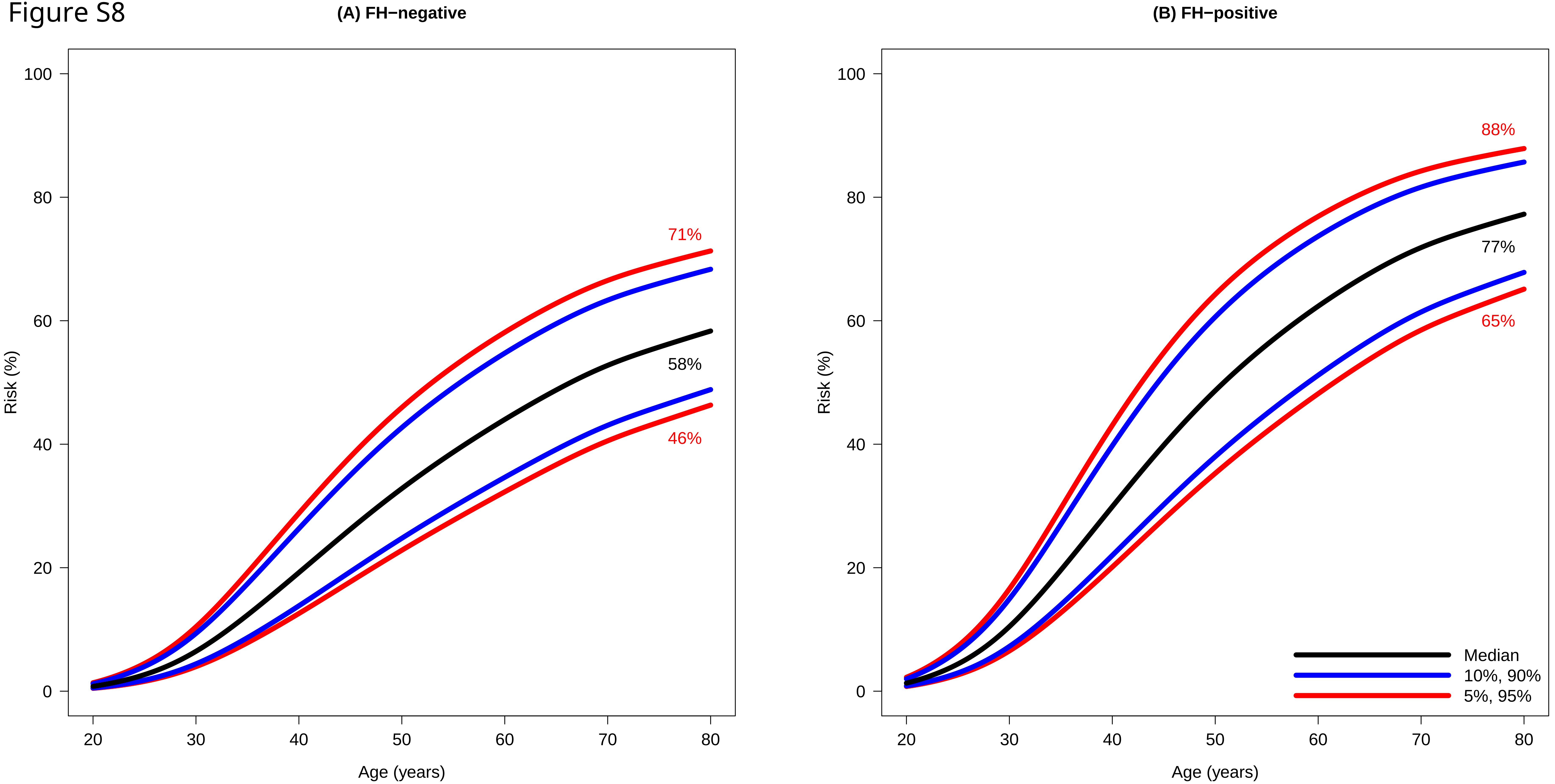
(A) FH−negative
(B) FH−positive
Risk (%)
Age (years)
100
80
60
40
20
0
20
30
40
50
60
70
80
71%
58%
46%
88%
77%
65%
Median
10%, 90%
5%, 95%

Figure S9

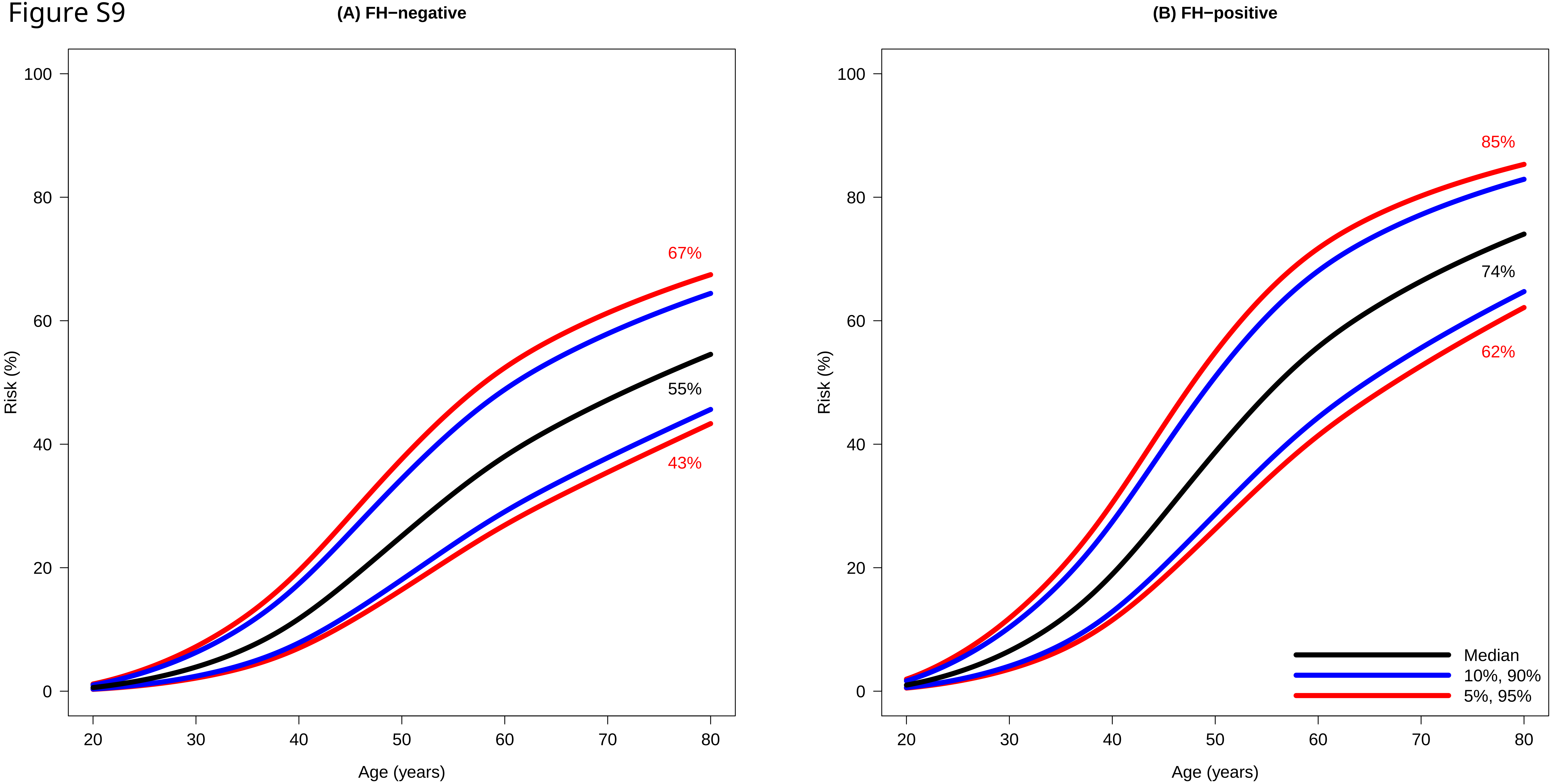
(A) FH−negative
(B) FH−positive
100
80
60
40
20
0
Risk (%)
20
30
40
50
60
70
80
Age (years)
67%
55%
43%
85%
74%
62%
Median
10%, 90%
5%, 95%
